# Association of longitudinal alcohol consumption trajectories with coronary heart disease: a meta-analysis of six cohort studies using individual participant data

**DOI:** 10.1186/s12916-018-1123-6

**Published:** 2018-08-22

**Authors:** Dara O’Neill, Annie Britton, Mary K. Hannah, Marcel Goldberg, Diana Kuh, Kay Tee Khaw, Steven Bell

**Affiliations:** 1 CLOSER, Department of Social Science, Institute of Education, University College London, London, UK; 2 Research Department of Epidemiology and Public Health, University College London, London, UK; 3 MRC/CSO Social and Public Health Sciences Unit, Institute of Health and Wellbeing, University of Glasgow, Glasgow, UK; 4 Inserm UMS 011, Villejuif, France and Paris Descartes University, Villejuif, France; 5 UK MRC Unit for Lifelong Health & Ageing at UCL, London, UK; 6 Cambridge Institute of Public Health, University of Cambridge, Cambridge, UK; 7 Department of Public Health and Primary Care, University of Cambridge, Cambridge, UK

**Keywords:** Alcohol, Coronary heart disease, IPD meta-analysis, Longitudinal design

## Abstract

**Background:**

Studies have shown that alcohol intake trajectories differ in their associations with biomarkers of cardiovascular functioning, but it remains unclear if they also differ in their relationship to actual coronary heart disease (CHD) incidence. Using multiple longitudinal cohort studies, we evaluated the association between long-term alcohol consumption trajectories and CHD.

**Methods:**

Data were drawn from six cohorts (five British and one French). The combined analytic sample comprised 35,132 individuals (62.1% male; individual cohorts ranging from 869 to 14,247 participants) of whom 4.9% experienced an incident (fatal or non-fatal) CHD event. Alcohol intake across three assessment periods of each cohort was used to determine participants’ intake trajectories over approximately 10 years. Time to onset for (i) incident CHD and (ii) fatal CHD was established using surveys and linked medical record data. A meta-analysis of individual participant data was employed to estimate the intake trajectories' association with CHD onset, adjusting for demographic and clinical characteristics.

**Results:**

Compared to consistently moderate drinkers (males: 1–168 g ethanol/week; females: 1–112 g ethanol/week), inconsistently moderate drinkers had a significantly greater risk of incident CHD [hazard ratio (HR) = 1.18, 95% confidence interval (CI) = 1.02–1.37]. An elevated risk of incident CHD was also found for former drinkers (HR = 1.31, 95% CI = 1.13–1.52) and consistent non-drinkers (HR = 1.47, 95% CI = 1.21–1.78), although, after sex stratification, the latter effect was only evident for females. When examining fatal CHD outcomes alone, only former drinkers had a significantly elevated risk, though hazard ratios for consistent non-drinkers were near identical. No evidence of elevated CHD risk was found for consistently heavy drinkers, and a weak association with fatal CHD for inconsistently heavy drinkers was attenuated following adjustment for confounding factors.

**Conclusions:**

Using prospectively recorded alcohol data, this study has shown how instability in drinking behaviours over time is associated with risk of CHD. As well as individuals who abstain from drinking (long term or more recently), those who are inconsistently moderate in their alcohol intake have a higher risk of experiencing CHD. This finding suggests that policies and interventions specifically encouraging consistency in adherence to lower-risk drinking guidelines could have public health benefits in reducing the population burden of CHD. The absence of an effect amongst heavy drinkers should be interpreted with caution given the known wider health risks associated with such intake.

**Trial registration:**

ClinicalTrials.gov, NCT03133689.

**Electronic supplementary material:**

The online version of this article (10.1186/s12916-018-1123-6) contains supplementary material, which is available to authorized users.

## Background

The relationship between alcohol consumption and coronary heart disease (CHD) is of scientific and public health interest, yet it remains a subject of debate. Studies have found evidence both for and against the possibility of an association [1, 2]. The concept of a potentially cardioprotective effect of moderate drinking compared to non-drinking or heavier consumption, termed the U/J-shaped curve, has been particularly controversial [3–5]. Some clinical evidence suggests that alcohol may affect different pathways thought to influence CHD risk, including hypertension [6, 7], body mass index (BMI) [8] and lipid levels [9, 10]. However, this purported effect continues to be disputed [11], which poses challenges in the formation of health-care policy and can hinder wider public understanding of the health impact of lifestyle choices.

Much of the discussion around the evidence base for the alcohol–CHD association has focussed on design limitations in observational studies, such as the failure to distinguish between non-drinkers and former drinkers [12, 13]. The decision to stop drinking could be influenced by the onset of ill health, and such sick quitters could potentially bias estimates of disease risk in lifelong abstainers if not analysed independently [14]. Studies have most commonly used single baseline measures of alcohol intake and that drinking behaviours can change over time has, therefore, not typically been reflected in the alcohol epidemiology literature [15, 16].

Recent efforts have been made to establish long-term trajectories of alcohol intake, enabling differentiation between patterns of drinking that fluctuate over time. Different trajectories have been found to have distinct patterns of association with intermediate markers of cardiovascular health, including carotid intima media thickness [17], pulse wave velocity [18] and inflammatory markers [19], but this work has yet to link these drinking typologies to CHD events directly. More commonly, studies with longitudinal assessments of drinking have used average intake, typically between only two measurement occasions, in evaluations of CHD risk [20], but such aggregation can mask consumption variation over time. The importance of capturing variability is evident from previous work that has shown how isolated episodes of heavy drinking can offset the potentially protective effects of moderate drinking [2]. Failure to account for stability in alcohol intake levels may bias risk estimates [21]. In the current study, we have used an alcohol intake trajectory approach, previously employed in the study of intermediate CHD markers [17–19], to address this research gap. We have drawn data from multiple cohort studies to investigate whether longitudinal trajectories of alcohol consumption differ in their association with total CHD incidence (fatal or non-fatal). Furthermore, since research has suggested that the cardioprotective effect of moderate drinking may be less evident with fatal CHD outcomes [22], particularly in comparison to heavier intake [23], a secondary aim of this work was to examine how the longitudinal trajectories are specifically associated with mortality due to CHD.

## Methods

### Sample, design and cohort selection

**Fig. 1:**
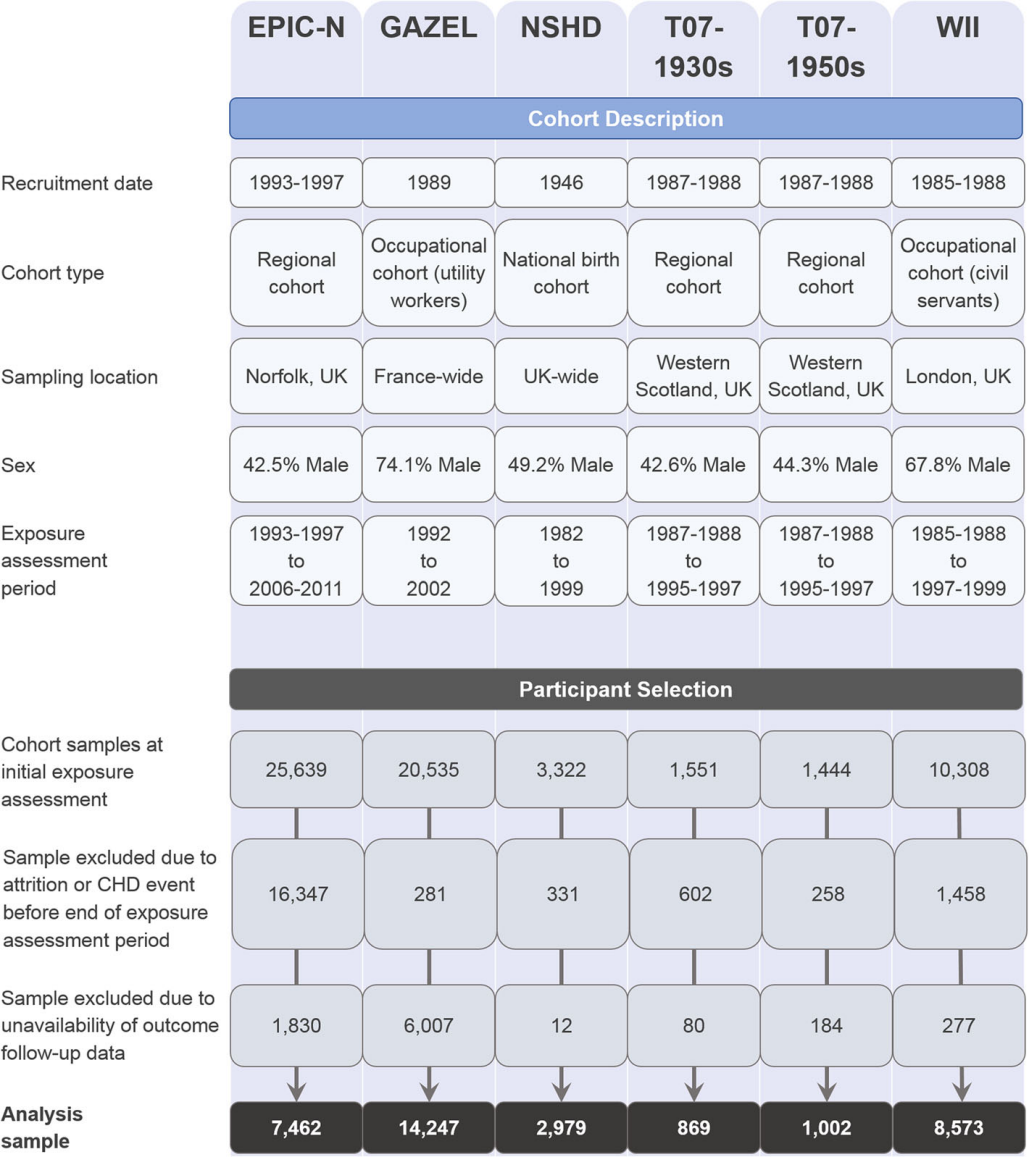
Cohort description and participant selection flowchart. CHD coronary heart disease, EPIC-N European Prospective Investigation of Cancer, Norfolk, GAZEL Gaz et Electricité, T07-1930s West of Scotland Twenty-07 Study 1930s, T07-1950s West of Scotland Twenty-07 Study 1950s, WII Whitehall II

Data were obtained from five British cohort studies: the European Prospective Investigation of Cancer, Norfolk Cohort (EPIC-N) [24]; the Medical Research Council’s National Survey of Health and Development 1946 (NSHD) [25]; West of Scotland Twenty-07: 1930s (T07-1930s) [26]; West of Scotland Twenty-07: 1950s (T07-1950s) [26] and Whitehall II (WII) [27]. Further data were obtained from an additional French cohort: Gaz et Electricité (GAZEL) [28]. Descriptions of each cohort are provided in Fig. 1 and complete cohort profiles are available via the above citations. The cohorts were chosen for their coverage of relevant variables and design similarity. They each included prospective alcohol intake data across three assessments covering an approximate 10-year interval, as well as pertinent covariate and verified CHD outcome data. Prior to commencement of the analysis, additional harmonisation was performed for all cohort datasets to maximise consistency in variable names and definitions. The study design was pre-registered on ClinicalTrials.gov (identifier NCT03133689), and a STROBE statement is provided in Additional file 1 (Section S1).

The combined dataset initially comprised 62,799 participant records in total (cohort-specific counts are provided in Fig. 1). The exposure variable was measured across three assessment points covering a decade on average, with the last assessment point treated as the study baseline from which time-to-event outcomes were measured. Of the initial sample, 19,277 participants were excluded due to attrition or having experienced a CHD event prior to the study baseline. A further 8390 participants were not included due to incomplete data linkage. Following these exclusion criteria (further details of which are provided in Fig. 1), a sample of 35,132 (62.1% male) participants remained and these comprised the analytic sample.

### Measures

#### Outcomes

The primary endpoint was CHD incidence, as ascertained from linked health records and survey data. Non-fatal CHD data were available for NSHD, but the time to event from the end of the exposure period was not ascertainable, so this study was omitted from the analysis of the incident CHD end point. Mortality due to CHD was examined as a secondary outcome in supplementary analyses, and all cohorts contributed data to this analysis. CHD events were previously coded using the codebook of the International Statistical Classification of Diseases and Related Health Problems (ICD) [29]: ICD-9: 410–414 Ischaemic heart disease and ICD-10: I20-I25 Ischaemic heart diseases. For the Twenty-07 cohorts, non-fatal CHD events were identified using the Royal College of General Practitioners’ codebook [30] (codes 1940, 1945 and 1950). Survival time was calculated for all participants as time (in years) between the end of the alcohol assessment period and date of CHD event, death from non-CHD causes, study dropout or last date of data linkage (study specific), whichever occurred first. Additional details of study enrolment and follow-up procedures are available in Additional file 1 (Section S10).

#### Exposure

Trajectories of self-reported alcohol intake were derived using weekly alcohol intake measurements collected across three consecutive waves of each cohort study. The resultant trajectories comprised a decade of follow-up on average. Standard drink definitions were used to define alcohol (ethanol) content in reported drinks (half-pints of beer or cider, small glasses of wine, and single servings of spirits): 8 g ethanol in the British cohort data and 10 g in the French GAZEL data [31, 32]. Reported consumption at each measurement occasion was categorised according to UK drinking guidelines at the time of data collection, which recommended a maximum intake level for lower-risk drinking of 168 g of ethanol a week for males and 112 g of ethanol a week for females [33]. Although recently published UK guidelines have proposed identical thresholds for males and females [34], the analytic report upon which they are based identified risk functions for both CHD morbidity and mortality that notably differed between males and females [35], particularly at higher levels of consumption [23]. The focus in the present study was on stability of adherence to lower-risk drinking guidelines over time, and we consequently categorised participants according to longitudinal profiles as defined in Table 1. Consistently moderate drinkers were used as the reference category [14]. Drinkers with inconsistent levels of alcohol intake were defined according to their modal intake, i.e. their most frequent level of intake. For example, where a participant had an equal number of heavy and moderate drinking periods, they were categorised as inconsistently heavy drinkers. This ensured that participants who occasionally drank heavily were not grouped with participants who consistently adhered to lower-risk drinking guidelines.

**Table 1:** Drinker type definitions with observed counts and percentages (within sex and overall)

Drinker type	Weekly alcohol intake	*N* (%)		
		Male	Female	Total
Consistent non-drinker	0 g at each wave of data collection	807 (4.6)	1335 (12.4)	2142 (7.5)
Former drinker	0 g at last wave but intake >0 g at any earlier wave	1831 (10.4)	2249 (21.0)	4080 (14.4)
Consistently moderate	Male: 1–168 g at each wave	7249 (41.0)	4161 (38.8)	11,410 (40.2)
	Female: 1–112 g at each wave			
Inconsistently moderate	Male: 1–168 g for most but not all waves	3599 (20.4)	2037 (19.0)	5636 (19.8)
	Female: 1–112 g for most but not all waves			
Consistently heavy	Male: >168 g at each wave	2216 (12.5)	349 (3.3)	2565 (9.0)
	Female: >112 g at each wave			
Inconsistently heavy	Male: >168 g for most but not all waves	1979 (11.2)	598 (5.6)	2577 (9.1)
	Female: >112 g for most but not all waves			

#### Covariates

Known demographic and lifestyle risk factors for CHD were selected for inclusion in the modelling, including sex and age. Socioeconomic position was defined using the participant’s occupational status, categorised as low (non-skilled or semi-skilled), intermediate (mid-level) or high (professional or executive) [36]. Smoking status was assessed, with participants categorised as current, ex- or non-smokers. To account for variability in the alcohol intake assessment interval, the time difference between initial and final assessments was calculated for each participant. Additional clinical data were obtained on BMI (measured as kg/m^2^) and self-reported high blood pressure or use of antihypertensive medication (yes/no). All of the covariates were assessed at the commencement of the follow-up period for CHD (the occasion of the third and final alcohol assessment), which we have defined as the current study’s baseline.

### Statistical analysis

Prior to undertaking inferential analyses, multiple imputation by chained equations was completed using the R ‘mice’ package (v2.30) to address missing covariate and exposure data. Altogether, 100 imputations were performed for both the incident and fatal CHD analyses, ensuring congruence between the imputation and substantive models. Outcome data with the Nelson–Aalen hazard [37] were used but not imputed.

The modelling was performed as individual participant data (IPD) meta-analyses, accounting for the clustering of participants within each cohort. Both one- and two-step approaches are available and can give comparable results under particular conditions [38]. However, the one-step approach, in which all data are analysed simultaneously with clustering incorporated as a random effect term, is thought to be less prone to bias in pooled effect estimates and standard errors [39] and to be the preferred approach where covariate adjustments are required or where inter-study heterogeneity may be present [40, 41]. Consequently, one-step IPD meta-analysis was performed using hierarchical (mixed effects) Cox regression modelling incorporating a random effect term for cohort membership with maximum likelihood estimations. Models were developed iteratively: an initial model accounting for age, sex and intake assessment interval (partially adjusted for confounding), followed by an extended model that additionally included smoking status and socioeconomic status covariates (maximally adjusted for confounding). Supplementary modelling extended the adjustment further, including potential mediators, to examine clinical pathways (maximally adjusted for confounding and mediation). Schoenfeld residuals were plotted to ascertain that the proportional hazards assumption had not been violated (available in Additional file 1: Section S2).

Given most existing work in this area has employed single one-off measures of alcohol intake, for comparative purposes, an initial IPD meta-analysis was undertaken in this study using participants’ final intake measurement prior to the outcome follow-up period (i.e. at this study’s baseline). This single measure categorisation allowed a distinction to be made between different intake levels (none, moderate or heavy), but not stability of intake over time or discontinuation of drinking. This analysis was followed by the modelling of the primary exposure, the longitudinal drinking trajectory categorisation. Additional stratified analyses were also completed to explore specific characteristics of the alcohol–CHD relationship. Research has suggested that the association of alcohol with cardiovascular risk may differ between older and younger populations [42], so age-stratified modelling of the longitudinal drinker typology was also performed (aged ≤55 vs >55 years at this study’s baseline). Further stratified analyses were undertaken to explore sex-specific effects. Finally, sensitivity analyses were conducted to determine the impact of modelling assumptions on this study’s main results.

The statistical analyses were performed in R (v3.4.1; R Foundation for Statistical Computing, Vienna, Austria). All statistical significance testing was two-tailed, using an inference threshold of *p* < 0.05.

## Results

### Sample characteristics

Descriptive statistics, for the overall sample and stratified by drinker type, are presented in Table 2. Additional descriptive statistics, stratified by cohort, are provided in Additional file 1 (Section S3). Statistics on data missingness are also reported in Table 2, and further detail is provided in Additional file 1 **(**Section S4).

**Table 2:** Descriptive results: overall sample

Variable	Level	Drinker type							Overall
		Consistent non-drinker	Former drinker	Consistently moderate drinker	Inconsistently moderate drinker	Consistently heavy drinker	Inconsistently heavy drinker	Unknown	
Record count, *N*		2142	4080	11,410	5636	2565	2577	6722	35,132
Age, Mean (SD)		58.9 (8.1)	61.9 (9.1)	59.7 (8.1)	57.1 (6.4)	58.0 (5.7)	57.9 (6.6)	58.6 (7.3)	59.1 (7.7)
Sex, *N* (%)	Male	807 (37.7)	1831 (44.9)	7249 (63.5)	3599 (63.9)	2216 (86.4)	1979 (76.8)	4140 (61.6)	21,821 (62.1)
	Female	1335 (62.3)	2249 (55.1)	4161 (36.5)	2037 (36.1)	349 (13.6)	598 (23.2)	2582 (38.4)	13,311 (37.9)
Smoker, *N* (%)	No	1351 (63.1)	2383 (58.4)	6933 (60.8)	3124 (55.4)	1218 (47.5)	1234 (47.9)	2280 (33.9)	18,523 (52.7)
	Current smoker	321 (15.0)	494 (12.1)	1008 (8.8)	764 (13.6)	540 (21.1)	473 (18.4)	1022 (15.2)	4622 (13.2)
	Ex-smoker	407 (19.0)	1129 (27.7)	3322 (29.1)	1630 (28.9)	759 (29.6)	825 (32.0)	1335 (19.9)	9407 (26.8)
	Unknown	63 (2.9)	74 (1.8)	147 (1.3)	118 (2.1)	48 (1.9)	45 (1.7)	2085 (31.0)	2580 (7.3)
Socioeconomic position, *N* (%)	High	510 (23.8)	1353 (33.2)	5454 (47.8)	2279 (40.4)	1178 (45.9)	1198 (46.5)	2400 (35.7)	14,372 (40.9)
	Intermediate	1104 (51.5)	1953 (47.9)	4924 (43.2)	2708 (48.0)	1184 (46.2)	1158 (44.9)	3005 (44.7)	16,036 (45.6)
	Low	512 (23.9)	733 (18.0)	980 (8.6)	612 (10.9)	190 (7.4)	207 (8.0)	1263 (18.8)	4497 (12.8)
	Unknown	16 (0.7)	41 (1.0)	52 (0.5)	37 (0.7)	13 (0.5)	14 (0.5)	54 (0.8)	227 (0.6)
BMI, mean (SD)		26.2 (4.9)	26.3 (4.4)	25.6 (3.5)	26.0 (3.9)	26.1 (3.3)	26.2 (3.7)	26.5 (4.1)	26 (3.9)
High blood pressure, *N* (%)	No	1498 (69.9)	2784 (68.2)	8409 (73.7)	4010 (71.1)	1790 (69.8)	1759 (68.3)	3376 (50.2)	23,626 (67.2)
	Yes	641 (29.9)	1292 (31.7)	2990 (26.2)	1620 (28.7)	774 (30.2)	815 (31.6)	1551 (23.1)	9683 (27.6)
	Unknown	3 (0.1)	4 (0.1)	11 (0.1)	6 (0.1)	1 (~ 0.0)	3 (0.1)	1795 (26.7)	1823 (5.2)
Intake interval, mean (SD)		11.4 (2.6)	11.8 (2.4)	11.4 (2.2)	11.2 (2.4)	10.7 (1.7)	11.1 (2.3)	12.6 (2.8)	11.5 (2.4)
CHD (all) during follow-up, *N* (%)	No	1803 (84.2)	3549 (87.0)	10,260 (89.9)	4775 (84.7)	2375 (92.6)	2229 (86.5)	4748 (70.6)	29,739 (84.6)
	Yes	129 (6.0)	250 (6.1)	560 (4.9)	264 (4.7)	98 (3.8)	107 (4.2)	310 (4.6)	1718 (4.9)
	Unknown	210 (9.8)	281 (6.9)	590 (5.2)	597 (10.6)	92 (3.6)	241 (9.4)	1664 (24.8)	3675 (10.5)
CHD (all) person years, mean (SD)		12.7 (4.5)	11.0 (4.7)	12.4 (4.4)	13.8 (3.7)	13.9 (3.4)	13.5 (3.8)	12.1 (4.5)	12.6 (4.3)
CHD (fatal) during follow-up, *N* (%)	No	2113 (98.6)	4031 (98.8)	11,328 (99.3)	5593 (99.2)	2549 (99.4)	2550 (99.0)	6630 (98.6)	34,794 (99.0)
	Yes	27 (1.3)	49 (1.2)	78 (0.7)	40 (0.7)	15 (0.6)	25 (1.0)	91 (1.4)	325 (0.9)
	Unknown	2 (0.1)	0 (0.0)	4 (~0.0)	3 (0.1)	1 (~ 0.0)	2 (0.1)	1 (~ 0.0)	13 (~ 0.0)
CHD (fatal) person years, mean (SD)		13.9 (4.2)	11.9 (4.6)	13.2 (4.3)	14.8 (3.2)	14.6 (3.1)	14.3 (3.5)	13.9 (4.0)	13.7 (4.1)

*BMI* body mass index, *kg/m2* kilogram per metre squared, *CHD* coronary heart disease, *SD* standard deviation, *N* count

Across the drinker types, mean age ranged from 57.1 years (standard deviation, SD = 6.4) for the inconsistently moderate drinkers to 61.9 (SD = 9.1) for the former drinkers. Heavy drinkers were most likely to be male (consistently heavy 86.4%; inconsistently heavy 76.8%), whereas abstainers were more likely to be female (consistent non-drinker 62.3%; former drinker 55.1%). Heavy drinkers had the highest proportion reporting past or current smoking (consistently heavy 50.7%; inconsistently heavy 50.4%). Consistently moderate drinkers were most likely to be of high socioeconomic position (47.8%), followed by both consistently and inconsistently heavy drinkers (45.9% and 46.5%). Conversely, consistent non-drinkers had the highest proportion in a low socioeconomic position (23.9%). BMI showed little variation between drinker types (all had means of 26 kg/m^2^). Known hypertension was least common amongst consistently moderate drinkers (26.2%) and most common amongst inconsistently heavy drinkers (31.6%) and former drinkers (31.7%). The mean assessment interval covered by the drinking trajectories was similar across all drinker types (range 10.7–11.8 years).

Crude outcome statistics are also provided in Table 2. In the pooled sample, 4.9% of participants experienced an incident CHD (fatal or non-fatal) event during the follow-up. This was lowest for consistently heavy drinkers (3.8%) and highest for former-drinkers (6.1%). The mean follow-up time was 12.6 years (SD = 4.3). In total, 397,264.4 person-years at risk were captured, with mean person-years varying from 11.0 years (former drinkers) to 13.9 years (consistently heavy drinkers). The overall CHD incidence rate was 4.3 CHD cases per 1000 person-years.

The proportion of individuals dying due to CHD during the follow-up was 0.9%. This varied between drinker types, from 0.6% for the consistently heavy group to 1.3% amongst consistent non-drinkers. The mean follow-up time was 13.7 years (SD = 4.1). In combination, 480,124.7 person-years were captured for this outcome, with the mean person-years again lowest for former drinkers (11.9 years) but highest for inconsistently moderate drinkers (14.8 years). The overall rate of fatal CHD was 0.7 cases per 1000 person-years.

### Single intake measure categorisation

**Fig. 2:**
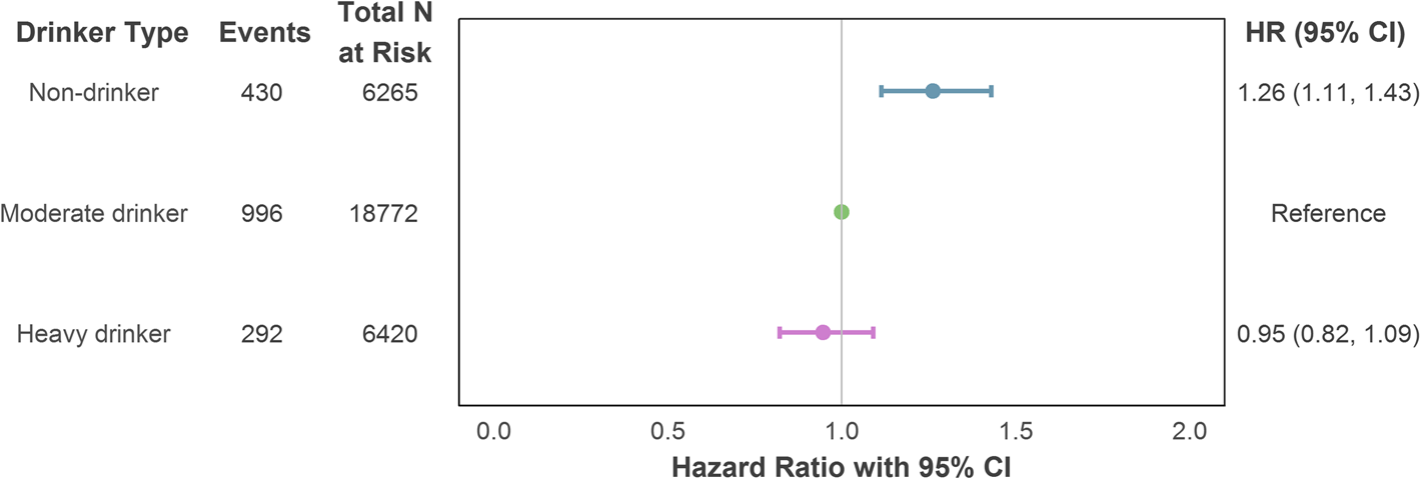
Association of drinker type (single intake measurement) with incident (fatal or non-fatal) CHD using maximal adjustment for confounding. Adjustment variables comprised age, sex (reference category: male), socioeconomic position (reference category: intermediate), smoker status (reference category: non-smoker) and intake assessment interval. CHD coronary heart disease, CI confidence interval, HR hazard ratio

In a series of hierarchical Cox regression models with alcohol intake defined according to a single intake measurement just prior to the outcome follow-up period, no discernible difference in incident CHD risk was observed between heavy and moderate drinkers. However, those who reported no intake at this most recent measurement point had an increased risk of CHD compared to those who drank but did so within the recommended limits [model maximally adjusted for confounding: hazard ratio (HR) = 1.26, 95% confidence interval (CI) = 1.11–1.43]. The estimates are illustrated in Fig. 2 and reported in full in Additional file 1 (Section S5a).

### Longitudinal intake trajectories

**Fig. 3:**
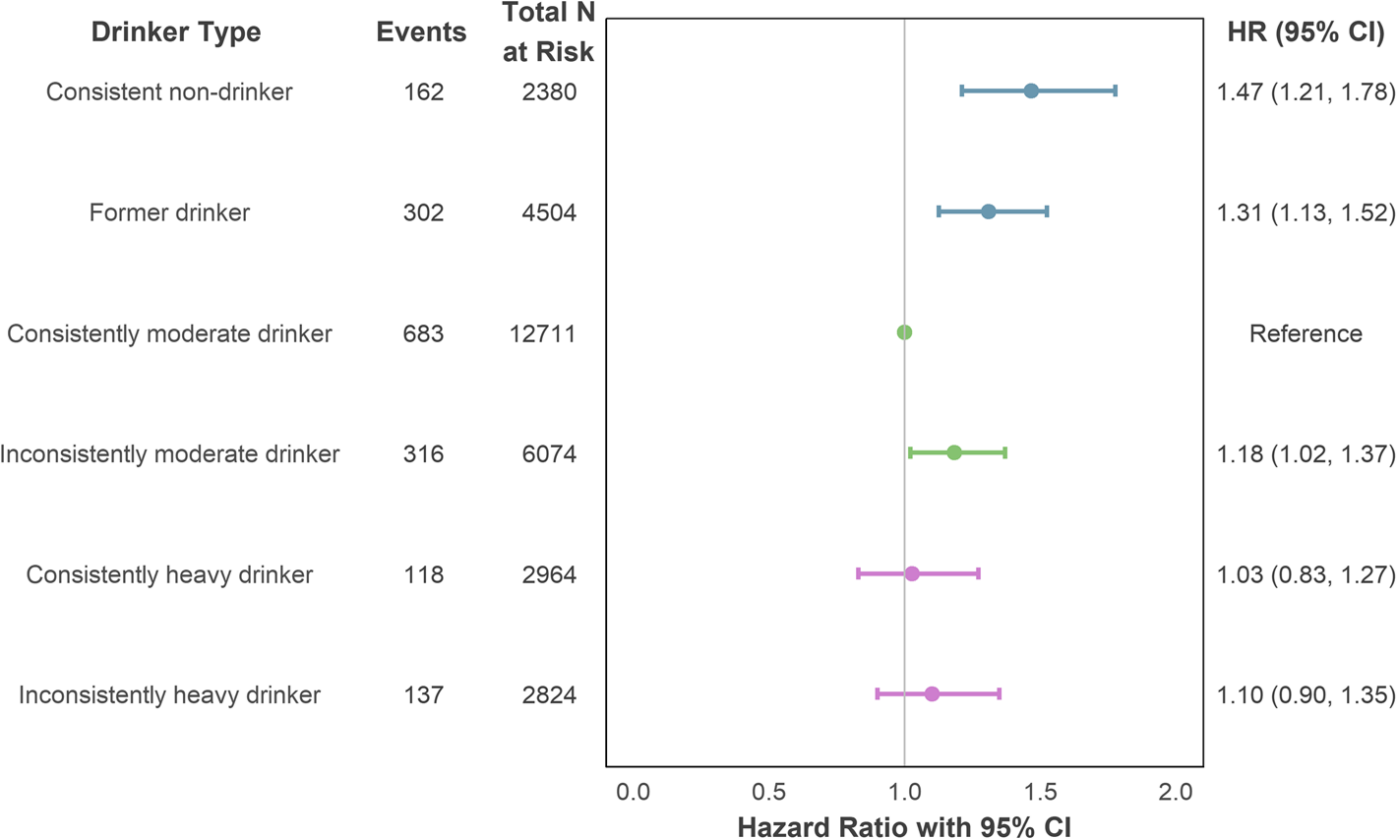
Association of drinker type (longitudinal intake measurement) with incident (fatal or non-fatal) CHD using maximal adjustment for confounding. Adjustment variables comprised age, sex (reference category: male), socioeconomic position (reference category: intermediate), smoker status (reference category: non-smoker) and intake assessment interval. CHD coronary heart disease, CI confidence interval, HR hazard ratio

When modelling overall CHD risk using the longitudinal intake typology with adjustment for age, sex and intake assessment interval, both consistent non-drinkers (HR = 1.51, 95% CI = 1.25–1.82) and former drinkers (HR = 1.35, 95% CI = 1.16–1.57) showed greater risk of incident CHD compared to participants who reported persistently moderate intake. A smaller but still significant effect was also found for inconsistently moderate drinkers (HR = 1.21, 95% CI = 1.04–1.40). The effects remained statistically significant after additional adjustment for smoking status and socioeconomic position (detailed in Fig. 3). No differences in risk for heavy drinking, consistent or otherwise, were found.

When potential mediators, BMI and hypertension, were included in the modelling, the drinker type effects were attenuated, with the effect for inconsistently moderate drinkers becoming non-significant (HR = 1.16, 95% CI = 1.00–1.34). Full details of the modelling steps are provided in Additional file 1 (Section S5a), including the associations of each covariate with CHD onset risk. Older age, male sex, history (current or past) of smoking, higher BMI and high blood pressure were all significantly associated with increased risk of CHD.

### Stratified analyses

**Fig. 4:**
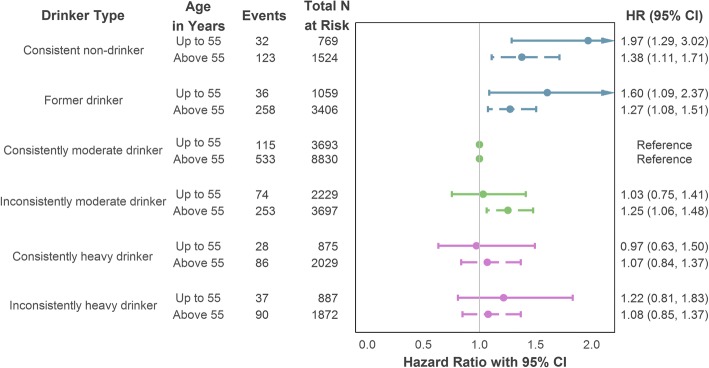
Age-stratified association of drinker type (longitudinal intake measurement) with incident (fatal or non-fatal) CHD using maximal adjustment for confounding. Adjustment variables comprised age, sex (reference category: male), socioeconomic position (reference category: intermediate), smoker status (reference category: non-smoker) and intake assessment interval. CHD coronary heart disease, CI confidence interval, HR hazard ratio

In age-stratified analyses of the longitudinal trajectory exposure, participants aged up to 55 years and those aged above showed comparable associations with the incident CHD outcome (visualised in Fig. 4). Consistent non-drinkers (aged ≤55: HR = 1.97, 95% CI = 1.29–3.02; aged > 55: HR = 1.38, 95% CI = 1.11–1.71) and former drinkers (aged ≤55: HR = 1.60, 95% CI = 1.09–2.37; aged > 55: HR = 1.27, 95% CI = 1.08–1.51) both demonstrated significantly greater CHD risk compared to consistently moderate drinkers. However, inconsistently moderate drinkers in the older age group also had an increased risk of incident CHD (HR = 1.25, 95% CI = 1.06–1.48), a finding not replicated in the younger subsample. Further details are provided in Additional file 1 (Section S6a).

**Fig. 5:**
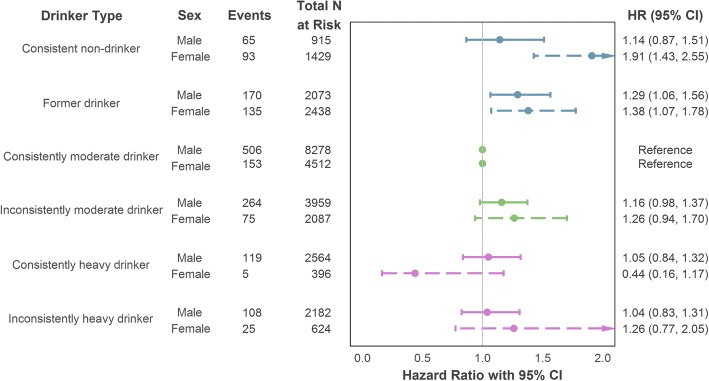
Sex-stratified association of drinker type (longitudinal intake measurement) with incident (fatal or non-fatal) CHD using maximal adjustment for confounding. Adjustment variables comprised age, socioeconomic position (reference category: intermediate), smoker status (reference category: non-smoker) and intake assessment interval. CHD coronary heart disease, CI confidence interval, HR hazard ratio

Further stratified analyses were performed to assess if the alcohol–CHD association differed by sex, again using the longitudinal intake categories (illustrated in Fig. 5). Amongst male participants, former drinkers were at significantly greater risk of incident CHD compared to consistently moderate drinkers after maximal adjustment for confounding factors (HR = 1.29, 95% CI = 1.06–1.56). After equivalent adjustment in the female stratum, both former drinkers (HR = 1.38, 95% CI = 1.07–1.78) and consistent non-drinkers (HR = 1.91, 95% CI = 1.43–2.55) showed increased risk compared to their consistently moderate counterparts. A full table of results is provided in Additional file 1 (Section S7a).

#### CHD mortality

When analyses were replicated using fatal CHD as the outcome, most results were comparable to those obtained when using all incident CHD events. For the longitudinal intake trajectories, and in contrast to the incident CHD analysis, inconsistently moderate drinkers did not have a greater CHD mortality risk compared to the consistently moderate reference group (HR = 1.04, 95% CI = 0.72–1.52). Only former drinkers had a significantly elevated risk of fatal CHD (HR = 1.54, 95% CI = 1.07–2.22) after maximal adjustment for confounding factors, but the HR for consistent non-drinkers was near identical (HR = 1.52, 95% CI = 0.97–2.38), implying that again both drinker types were at elevated risk of fatal CHD. Inconsistently heavy drinkers did show some evidence of having an increased risk of experiencing a fatal CHD event in the lesser-adjusted model (HR = 1.53, 95% CI = 0.99–2.37), but it did not achieve statistical significance and was attenuated after additional adjustment for smoking status and socioeconomic status (HR = 1.36, 95% CI = 0.87–2.11). Full model details are provided in Additional file 1 (Section S5b).

Age-stratified analyses revealed similar patterns of association as with the pooled (non-stratified) analysis. In sex-stratified analyses, however, some differences were observed, with only female consistent non-drinkers having an elevated risk of fatal CHD after adjustment for covariates (HR = 2.62, 95% CI = 1.25–5.49). Additional detail on age-stratified and sex-stratified analyses are included in Additional file 1 (Sections S6b and S7b, respectively).

#### Sensitivity analyses

As the GAZEL cohort was the only non-UK data source included in this study, the longitudinal modelling was replicated with this cohort’s data omitted to verify that its inclusion did not introduce bias. Results obtained using only UK data sources were essentially unchanged from the findings attained when all cohorts were included (details are provided in Additional file 1: Section S8). To identify the impact of the imputation model implemented in the primary analyses, the modelling was also performed using complete case data only. The point estimates and significance of effects were essentially unchanged from the imputed data modelling (see Additional file 1: Section S9).

## Discussion

In this study, we utilised prospectively collected longitudinal data on alcohol consumption from six cohorts to examine the association of 10-year drinking trajectories and risk of developing and/or dying from CHD. Through iterative modelling that accounted for heterogeneity across the datasets and potential confounders of the alcohol–CHD association, our work has shown that incident CHD risk is significantly higher amongst both non-drinkers and former drinkers compared to drinkers who always adhered to lower-risk intake guidelines. We have also demonstrated that the stability of such adherence is pertinent. Participants who mostly drank moderately, but not persistently so, had greater risk of incident CHD compared to their consistently moderate drinking counterparts. In terms of CHD mortality, former drinkers and consistent non-drinkers were again found to be at higher risk, although the effect for the persistent abstainers was somewhat attenuated after adjustment for smoking status and socioeconomic status. We found no evidence that heavy drinking was associated with risk of CHD, and reasons for this are discussed below. Overall, the findings from this study support the notion of a cardioprotective effect of moderate alcohol intake relative to non-drinking. However, crucially, stability in the level of alcohol consumption over time appears to be an important modifier of this association.

The use of repeat measurements of alcohol consumption in lieu of a one-time assessment has enabled us to measure the stability of consumption over time and to address the call for research on the role of intake trajectories in CHD onset [43]. Through this approach, we have demonstrated how intermittent adherence to lower-risk drinking guidelines, i.e. an inconsistently moderate intake, is associated with an increased risk of incident CHD. This provides some support for the proposal that variability in alcohol intake levels can offset the potential protective effects of moderate drinking [2, 20]. An association was found between inconsistently heavy drinkers and fatal CHD, although the wide confidence bounds and weakening of the association following maximal adjustment for confounding factors limits interpretation of this effect. It may be that unstable drinking patterns reflect wider lifestyle changes across the life course, and possibly even the impact of periods of ill health or life stress. The effects were further attenuated when adjustment was made for clinical characteristics, namely BMI and hypertension, suggesting that these may both act as potential pathways through which unstable drinking trajectories are associated with CHD. The impact of BMI could also reflect the role of other lifestyle choices, such as diet and exercise.

Access to prospectively recorded alcohol intake data across multiple assessment times has also allowed the current study to distinguish recent abstainers from longer-term non-drinkers in a manner that helps reduce the potential for recall bias. Such bias can occur where drinking behaviour is retrospectively measured at a single time point [44], a technique commonly used in alcohol epidemiology research. In line with the sick-quitter hypothesis [14], former drinkers were found in the present study to have an elevated risk of both incident and fatal CHD. These effects were attenuated following adjustment for the clinical covariates, suggesting that poor health may explain former drinkers’ increased likelihood of developing CHD and perhaps may even have motivated the decision to abstain itself. Consistent non-drinkers, however, did also have a significant risk of incident CHD after adjustment for potential confounders, and although the error bounds were wider, their CHD mortality estimate was equivalent to that of former drinkers, implying that short- and long-term abstinence are both associated with an increased risk of CHD.

Despite our finding of parity in CHD risk amongst non-drinkers and former drinkers in the pooled sample analyses, previous research has suggested that there may be age-dependent differences in this association. However, this observation was based on studies in which abstinence was determined retrospectively from a single baseline assessment [42], in contrast to the repeated measures design used in the current study. When we stratified our sample by age, the associations between both abstainer groups and incident CHD risk was comparable for younger (≤55 years) and older (>55 years) participants. As similar results were also observed for risk of fatal CHD, our findings challenge the argument that there are age-dependent differences between long-term and more recent abstainers, yet the wide confidence bounds around the fatal CHD risk estimates for those aged 55 or below arguably restricts such inferences. A divergence between the age groups was found for inconsistently moderate drinkers. Such drinkers in the older subsample had a significantly elevated risk of incident CHD, an effect that was not evident in the younger group. Older participants may have been more likely to experience lifestyle changes that influenced their drinking habits. Retirement, for example, is known to co-occur with increases in alcohol intake [45, 46], particularly amongst existing drinkers [47].

It has also been suggested that the J-shaped association between alcohol consumption and CHD may be more pronounced in women than men [23], a theory that our study supports in part. Whilst both male and female former drinkers had significantly increased risk of incident CHD, only female consistent non-drinkers showed such an elevated risk. Female non-drinkers (both long term and more recent abstainers) were similarly at risk of fatal CHD, even after maximal adjustment for confounding factors. Research has also suggested that alcohol intake may increase oestrogen levels in women, which in turn act as a protective factor against CHD [48]. Male former drinkers also showed significantly greater risk of CHD mortality than consistently moderate drinkers after accounting for age and other characteristics, but this difference was attenuated once the estimates were adjusted for lifestyle behaviours such as smoking. This suggests that these additional covariates may play a greater role than drinking in the occurrence of fatal CHD events for males. Previous literature has proposed that smoking can offset any alcohol-related differences in CHD mortality risk amongst men [49].

In the present study, no association with CHD risk was found for consistently heavy drinkers. Stable patterns of heavy drinking may reflect continued good health across the assessment interval [50], the converse of the sick quitter type. Statistically significant associations between high levels of alcohol intake and CHD onset risk have been observed in some previous research [21, 51], but not persistently so [52–54]. Although our study identified heavy drinkers across all cohorts, only a limited number were in the female sample, potentially limiting statistical power in their analysis, and by extension, in the non-stratified analysis. This issue of small counts for female heavy drinkers has similarly constrained earlier work in this area [1]. Particularly heavy drinkers may be under-represented in the datasets utilised in this study, which could have biased downwards the estimate of association between heavy intake and cardiovascular risk. If further data are available, it may be possible to explore alternative intake thresholds and validate the present study’s findings. Similarly, additional data may enable the disaggregation of CHD phenotypes, which could provide more nuanced insights into how heavy drinking is associated with different variants of the disease [55]. Consequently, the interpretation of the absence of an effect amongst heavy drinkers in the current study should be done cautiously, particularly in light of the known wider health impact of heavy alcohol intake levels [56].

There are additional limitations to our study that warrant consideration. For example, selection bias may have occurred [57], in which participants dropped out of the cohort studies before the outcome assessment period. It is possible that some heavy drinkers could have experienced adverse health outcomes at a younger age and discontinued their research participation. Particularly heavy drinkers are already known to be under-sampled in population-level surveys [32, 58], so caution is required in drawing inferences about such elevated intake levels. Similarly, information on alcohol intake prior to the exposure assessment period was not consistently available, so the long-term abstainers modelled in this current study may include some participants who ceased drinking prior to recruitment. Given that the current work included only cohort studies for which we had access to individual-level data, the concept of availability bias [59] is also pertinent. Access to additional datasets may help further validate our findings. Such increased sample sizes would also permit more detailed examination than was possible in the current study into the intake variance that occurs amongst drinkers who are inconsistent in their adherence to lower-risk drinking guidelines. Relatedly, the identification of drinking trajectories in the present study was based on drinking volume only and so we were not equipped to look at the role of episodic heavy drinking [60]. Further clarification of the alcohol–CHD association may be achieved where sufficient data are available on other characteristics of consumption, such as drinking frequency. All cohorts included in the current study used self-report for determining alcohol intake; although this is vulnerable to estimation errors, research has shown that drinking data collected through this method remains valid and reliable [44, 61]. A further design consideration in interpreting the current study’s results is the harmonisation of data across the different cohort datasets. Establishing equivalent variable definitions in the harmonisation of data constrains the level of detail and raises the possibility of residual confounding. For example, it was not possible to establish a more nuanced smoking variable due to data availability and so there is a possibility of residual confounding by smoking intensity. Relatedly, although an equal number of intake measurements was used across cohorts to establish intake trajectories, the observed time intervals varied (see Section S2 of Additional file 1). While adjustment was made through inclusion of assessment interval length in the regression modelling, it remains possible that limitations in the cohort data harmonisation may have introduced bias. Although country-specific drink conversions were used to calculate alcohol intake [31], there remains potential differences between GAZEL and the other cohorts, such as the possible influence of dietary differences for which residual confounding could also have occurred [62]. The French paradox, for example, implies that that there is an inverse relationship between saturated fat intake and CHD onset risk that is specific to France [63], a relationship in which alcohol debatably plays a role [64]. However, sensitivity analyses showed that the exclusion of GAZEL data did not modify the current study findings. Moreover, the use throughout this study of mixed-effects modelling has helped account for data clustering and thereby helped improve the validity of the results obtained.

## Conclusions

In summary, the present study has utilised longitudinal alcohol intake data pooled from multiple cohort sources to establish trajectories of drinking behaviour and assess their association with risk of incident and fatal CHD. The study has demonstrated that recent and more long-term abstainers are at elevated risk of developing CHD, although the effect for persistent abstainers may be confined to females only. The trajectory approach used in this work has also enabled us to show that stability of alcohol intake levels amongst those who do not abstain is pertinent to risk of CHD onset. Drinkers who mostly, but inconsistently, adhered to moderate drinking levels, particularly if aged over 55 years, were found to have elevated risk of incident CHD. There was also some indication that variability in drinking levels amongst heavier drinkers were associated with increased likelihood of CHD mortality, although that effect was attenuated by adjustment for other demographic and lifestyle characteristics. No evidence of elevated risk amongst consistently heavy drinkers was found but this was potentially attributable to under-representation of such drinkers in the sampled data. These findings, nonetheless, illustrate that longitudinal alcohol trajectories have added utility in identifying at-risk drinker types beyond what is possible with single assessments of alcohol consumption. Our findings provide additional insight into the potential cardioprotective effect of moderate alcohol intake, and indicate that consistency of intake levels is a relevant consideration in cardiovascular risk assessments, and in related health education efforts.

## Additional file

Additional file 1: Supplementary Materials. (PDF 3002 kb)

## Abbreviations

BMI: Body mass index
CHD: Coronary heart disease
CI: Confidence interval
EPIC-N: European Prospective Investigation of Cancer, Norfolk
GAZEL: Gaz et Electricité
HR: Hazard ratio
ICD: International Statistical Classification of Diseases and Related Health Problems
IPD: Individual participant data
NSHD: Medical Research Council’s National Survey of Health and Development 1946
SD: Standard deviation
T07-1930s: West of Scotland Twenty-07 Study 1930s
T07-1950s: West of Scotland Twenty-07 Study 1950s
WII: Whitehall II
