# Supplementary material for: Association of longitudinal alcohol consumption trajectories with coronary heart disease: a meta-analysis of six cohort studies using individual participant data
Source: BMC Med. 2018 Aug 22;16:124. doi: 10.1186/s12916-018-1123-6 (PMC6103865; doi:10.1186/s12916-018-1123-6)
Supplement: Supplementary file 1 — Supplementary Materials. (PDF 3002 kb) [file 12916_2018_1123_MOESM1_ESM.pdf]

Supplementary Materials

Contents

S1. STROBE Statement 2

S2. Schoenfeld Residuals 4

S3. Descriptive Results: Stratified by Cohort 6

S4. Missing Data Summary 8

S5. Hierarchical Cox Regression Modelling Output 9

S6. Age-Stratified Results: Longitudinal Intake Typology 15

S7. Sex-Stratified Results: Longitudinal Intake Typology 20

S8. Fixed Effects Plots: UK Cohorts Only 28

S9. Fixed Effects Plots: Complete Case Data 30

S10. Study Enrolment and Follow-up Procedures 32

S1. STROBE Statement

STROBE Statement—Checklist of items that should be included in reports of ***cohort studies***

	Item No	Recommendation	
**Title and abstract**	1	(*a*) Indicate the study's design with a commonly used term in the title or the abstract	☑
		(*b*) Provide in the abstract an informative and balanced summary of what was done and what was found	☑
**Introduction**			
Background/rationale	2	Explain the scientific background and rationale for the investigation being reported	☑
Objectives	3	State specific objectives, including any prespecified hypotheses	☑
**Methods**			
Study design	4	Present key elements of study design early in the paper	☑
Setting	5	Describe the setting, locations, and relevant dates, including periods of recruitment, exposure, follow-up, and data collection	☑
Participants	6	(*a*) Give the eligibility criteria, and the sources and methods of selection of participants. Describe methods of follow-up	☑
		(*b*) For matched studies, give matching criteria and number of exposed and unexposed	n/a
Variables	7	Clearly define all outcomes, exposures, predictors, potential confounders, and effect modifiers. Give diagnostic criteria, if applicable	☑
Data sources/ measurement	8*	For each variable of interest, give sources of data and details of methods of assessment (measurement). Describe comparability of assessment methods if there is more than one group	☑
Bias	9	Describe any efforts to address potential sources of bias	☑
Study size	10	Explain how the study size was arrived at	☑
Quantitative variables	11	Explain how quantitative variables were handled in the analyses. If applicable, describe which groupings were chosen and why	☑
Statistical methods	12	(*a*) Describe all statistical methods, including those used to control for confounding	☑
		(*b*) Describe any methods used to examine subgroups and interactions	☑
		(*c*) Explain how missing data were addressed	☑
		(*d*) If applicable, explain how loss to follow-up was addressed	☑
		(*e*) Describe any sensitivity analyses	☑
**Results**			
Participants	13*	(a) Report numbers of individuals at each stage of study—eg numbers potentially eligible, examined for eligibility, confirmed eligible, included in the study, completing follow-up, and analysed	☑
		(b) Give reasons for non-participation at each stage	☑
		(c) Consider use of a flow diagram	☑

Descriptive data	14*	(a) Give characteristics of study participants (eg demographic, clinical, social) and information on exposures and potential confounders	☑
		(b) Indicate number of participants with missing data for each variable of interest	☑
		(c) Summarise follow-up time (eg, average and total amount)	☑
Outcome data	15*	Report numbers of outcome events or summary measures over time	☑
Main results	16	(*a*) Give unadjusted estimates and, if applicable, confounder-adjusted estimates and their precision (eg, 95% confidence interval). Make clear which confounders were adjusted for and why they were included	☑
		(*b*) Report category boundaries when continuous variables were categorized	☑
		(*c*) If relevant, consider translating estimates of relative risk into absolute risk for a meaningful time period	☑
Other analyses	17	Report other analyses done—eg analyses of subgroups and interactions, and sensitivity analyses	☑
**Discussion**			
Key results	18	Summarise key results with reference to study objectives	☑
Limitations	19	Discuss limitations of the study, taking into account sources of potential bias or imprecision. Discuss both direction and magnitude of any potential bias	☑
Interpretation	20	Give a cautious overall interpretation of results considering objectives, limitations, multiplicity of analyses, results from similar studies, and other relevant evidence	☑
Generalisability	21	Discuss the generalisability (external validity) of the study results	☑
**Other information**			
Funding	22	Give the source of funding and the role of the funders for the present study and, if applicable, for the original study on which the present article is based	☑

*Give information separately for exposed and unexposed groups.

Note: An Explanation and Elaboration article discusses each checklist item and gives methodological background and published examples of transparent reporting. The STROBE checklist is best used in conjunction with this article (freely available on the Web sites of PLoS Medicine at http://www.plosmedicine.org/, Annals of Internal Medicine at http://www.annals.org/, and Epidemiology at http://www.epidem.com/). Information on the STROBE Initiative is available at http://www.strobe-statement.org.

S2. Schoenfeld Residuals

Schoenfeld Residuals: Incident CHD (Fatal & Non-Fatal) for Imputed Dataset Models

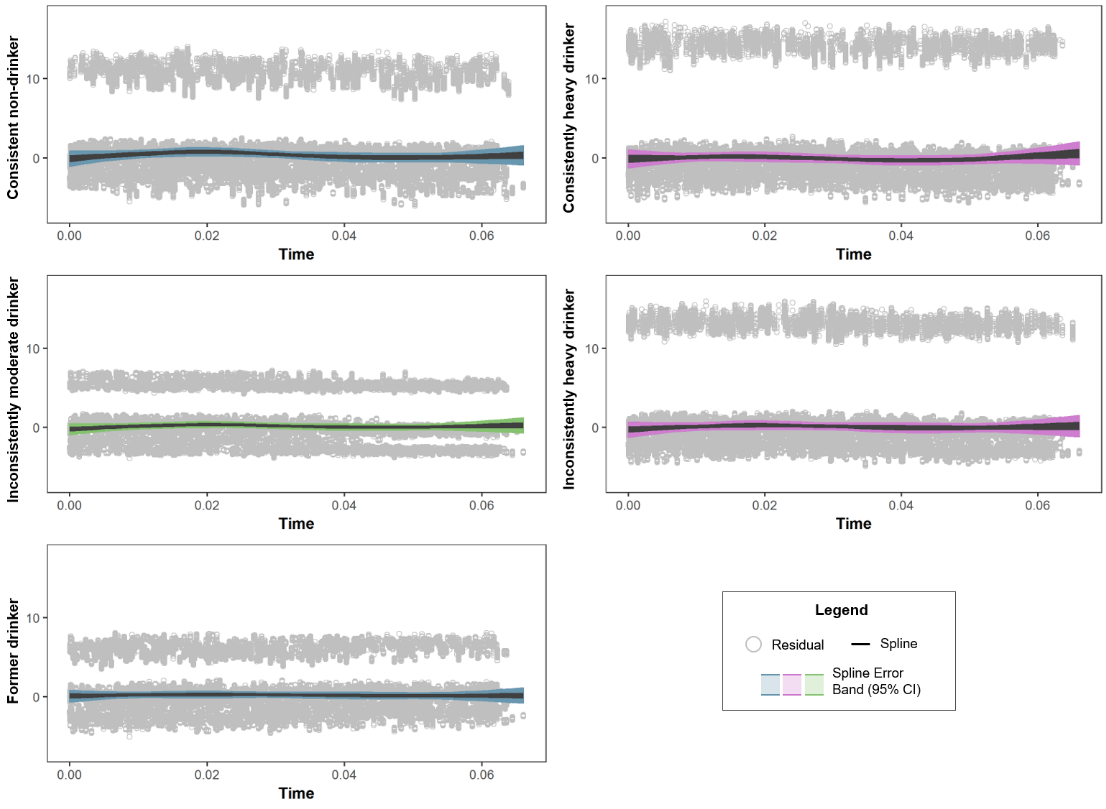

Schoenfeld Residuals: Incident CHD (Fatal & Non-Fatal) for Complete Case Dataset Models

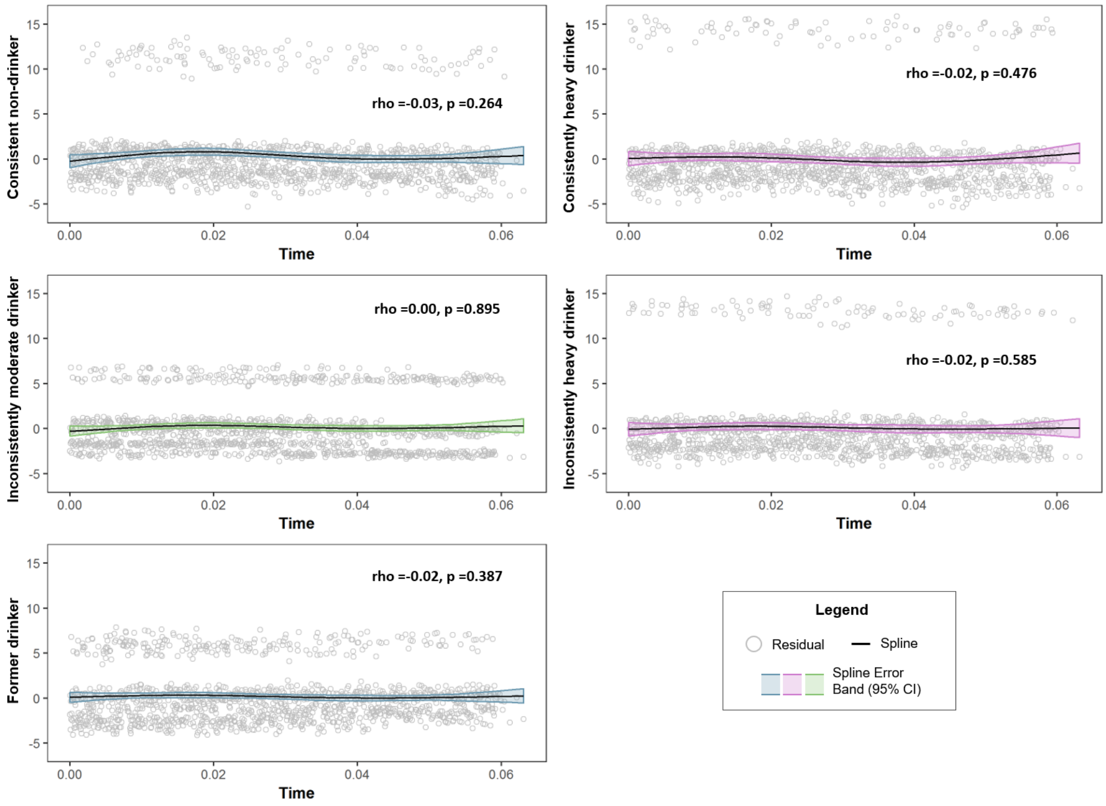

Schoenfeld Residuals: Fatal CHD for Imputed Dataset Models

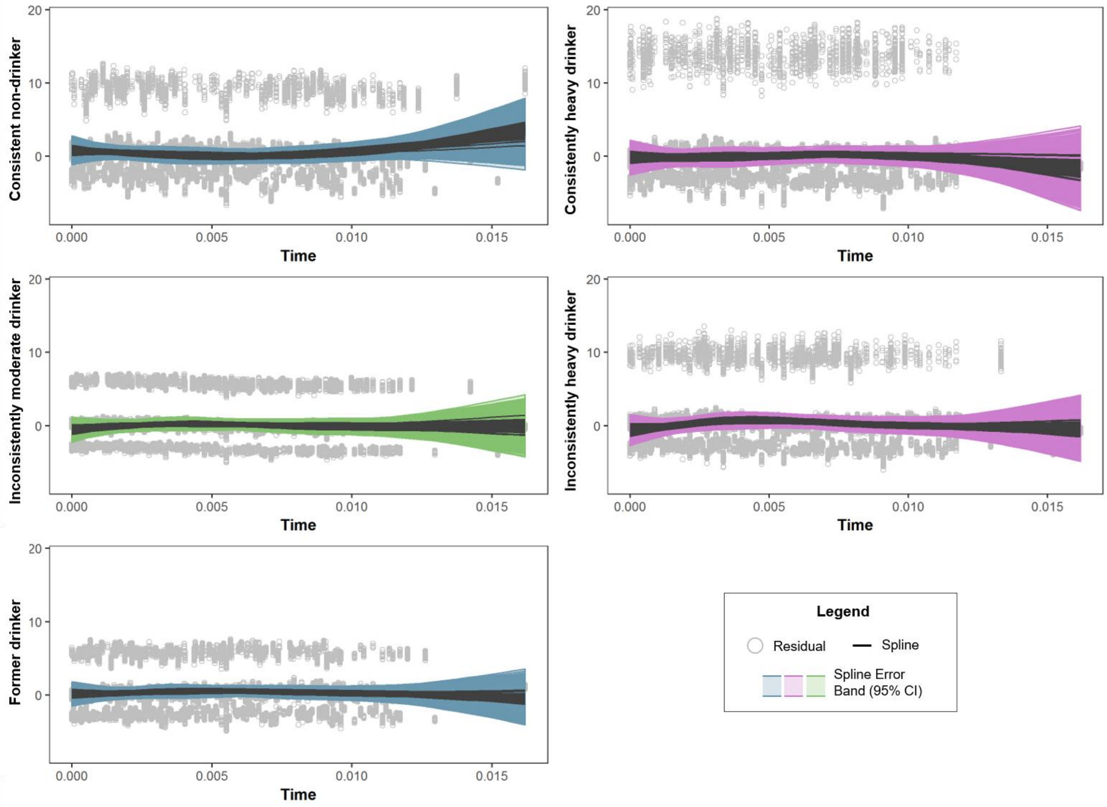

Schoenfeld Residuals: Fatal CHD for Complete Case Dataset Models

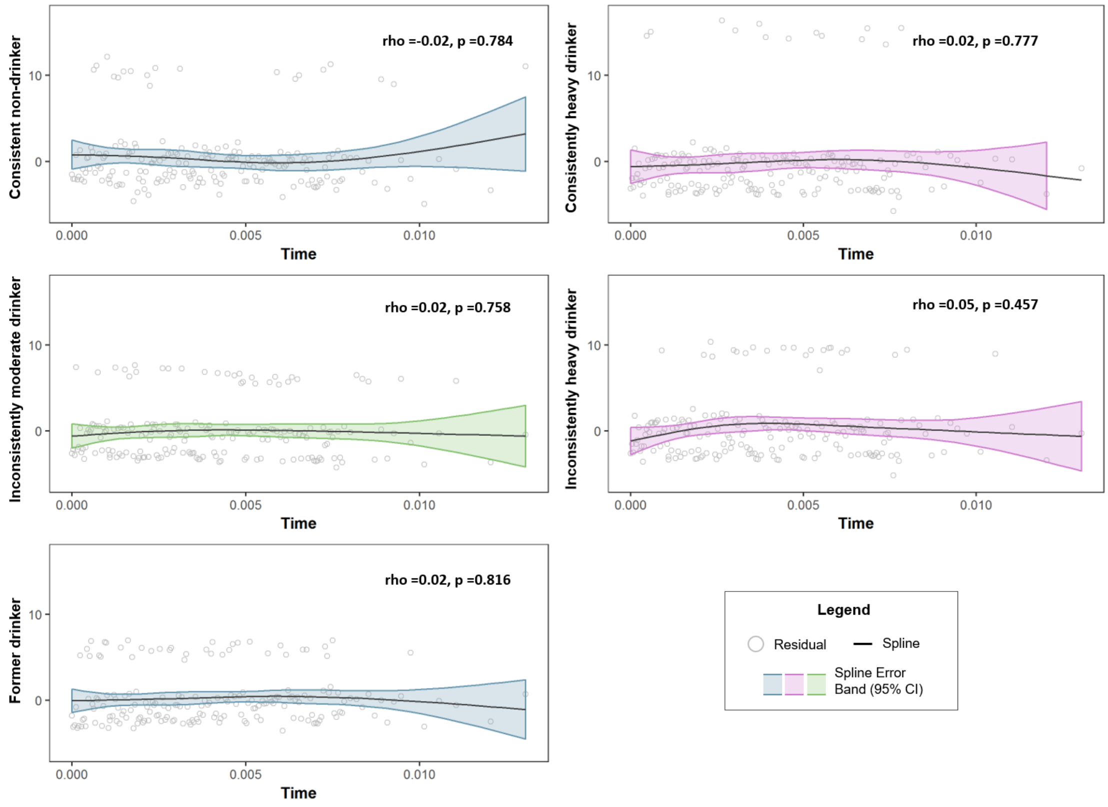

S3. Descriptive Results: Stratified by Cohort

Variable	Level	EPIC-N	GAZEL	NSHD (1946)	T07 (1930s)	T07 (1950s)	WII
Record Count, N		7462	14247	2979	869	1002	8573
Age, Mean (SD)		68.3 (8.0)	57.4 (3.5)	53.3 (1.1)	64.4 (1.2)	45.2 (1.2)	55.7 (6.0)
Sex, N (%)	Male	3172 (42.5)	10560 (74.1)	1466 (49.2)	370 (42.6)	444 (44.3)	5809 (67.8)
	Female	4290 (57.5)	3687 (25.9)	1513 (50.8)	499 (57.4)	558 (55.7)	2764 (32.2)
	Unknown	0 (0.0)	0 (0.0)	0 (0.0)	0 (0.0)	0 (0.0)	0 (0.0)
Smoker, N (%)	No	3753 (50.3)	9960 (69.9)	765 (25.7)	313 (36.0)	416 (41.5)	3316 (38.7)
	Current smoker	330 (4.4)	1869 (13.1)	1092 (36.7)	272 (31.3)	345 (34.4)	714 (8.3)
	Ex-smoker	3273 (43.9)	1862 (13.1)	1065 (35.8)	278 (32.0)	236 (23.6)	2693 (31.4)
	Unknown	106 (1.4)	556 (3.9)	57 (1.9)	6 (0.7)	5 (0.5)	1850 (21.6)
Socioeconomic Position, N (%)	High	3711 (49.7)	5064 (35.5)	1454 (48.8)	295 (33.9)	509 (50.8)	3339 (38.9)
	Intermediate	2693 (36.1)	7740 (54.3)	1106 (37.1)	398 (45.8)	372 (37.1)	3727 (43.5)
	Low	989 (13.3)	1318 (9.3)	386 (13.0)	176 (20.3)	121 (12.1)	1507 (17.6)
	Unknown	69 (0.9)	125 (0.9)	33 (1.1)	0 (0.0)	0 (0.0)	0 (0.0)
BMI, Mean (SD)		25.7 (3.6)	25.8 (3.6)	27.4 (4.8)	26.7 (4.4)	26.4 (4.6)	26.1 (3.9)
High Blood Pressure, N (%)	No	5283 (70.8)	10411 (73.1)	989 (33.2)	717 (82.5)	948 (94.6)	5278 (61.6)
	Yes	2179 (29.2)	3836 (26.9)	1990 (66.8)	152 (17.5)	54 (5.4)	1472 (17.2)
	Unknown	0 (0.0)	0 (0.0)	0 (0.0)	0 (0.0)	0 (0.0)	1823 (21.3)
Drinker Type, N (%)	Consistent non-drinker	422 (5.7)	798 (5.6)	210 (7.0)	118 (13.6)	87 (8.7)	507 (5.9)
	Former drinker	1689 (22.6)	1341 (9.4)	281 (9.4)	93 (10.7)	93 (9.3)	583 (6.8)
	Consistently moderate drinker	3270 (43.8)	4428 (31.1)	590 (19.8)	189 (21.7)	258 (25.7)	2675 (31.2)
	Inconsistently moderate drinker	527 (7.1)	2673 (18.8)	597 (20.0)	141 (16.2)	176 (17.6)	1522 (17.8)
	Consistently heavy drinker	226 (3.0)	1642 (11.5)	92 (3.1)	23 (2.6)	45 (4.5)	537 (6.3)
	Inconsistently heavy drinker	300 (4.0)	1380 (9.7)	241 (8.1)	41 (4.7)	74 (7.4)	541 (6.3)
	Unknown	1028 (13.8)	1985 (13.9)	968 (32.5)	264 (30.4)	269 (26.8)	2208 (25.8)
Intake Interval, Mean (SD)		12.9 (1.9)	10.0 (0.1)	17.0 (0.0)	8.2 (1.0)	9.1 (1.0)	11.2 (0.8)
CHD (All) During Follow-up, N (%)	No	6868 (92.0)	14067 (98.7)	0 (0.0)	745 (85.7)	961 (95.9)	7098 (82.8)
	Yes	594 (8.0)	180 (1.3)	0 (0.0)	124 (14.3)	41 (4.1)	779 (9.1)
	Unknown	0 (0.0)	0 (0.0)	2979 (100.0)	0 (0.0)	0 (0.0)	696 (8.1)
CHD (All) Person Years, Mean (SD)		6.9 (2.2)	14.8 (1.4)	n/a	14.2 (5.5)	17.6 (2.8)	13.3 (4.2)
CHD (Fatal) During Follow-up, N (%)	No	7396 (99.1)	14191 (99.6)	2946 (99.0)	820 (94.4)	991 (98.9)	8450 (98.7)
	Yes	66 (0.9)	56 (0.4)	31 (1.0)	49 (5.6)	11 (1.1)	112 (1.3)

CHD (Fatal) Person Years, Mean (SD)	7.2 (1.9)	14.9 (1.1)	14.5 (2.1)	15.0 (5.1)	17.9 (2.4)	16.4 (2.9)

Note: N=Count; SD=Standard deviation; kg/m^2=Kilogram per metre squared; BMI=Body mass index; CHD=Coronary heart disease

S4. Missing Data Summary

The following plot summarises the proportion of missing data in each variable included in the modelling, alongside a list of the missing data patterns observed in the overall sample.

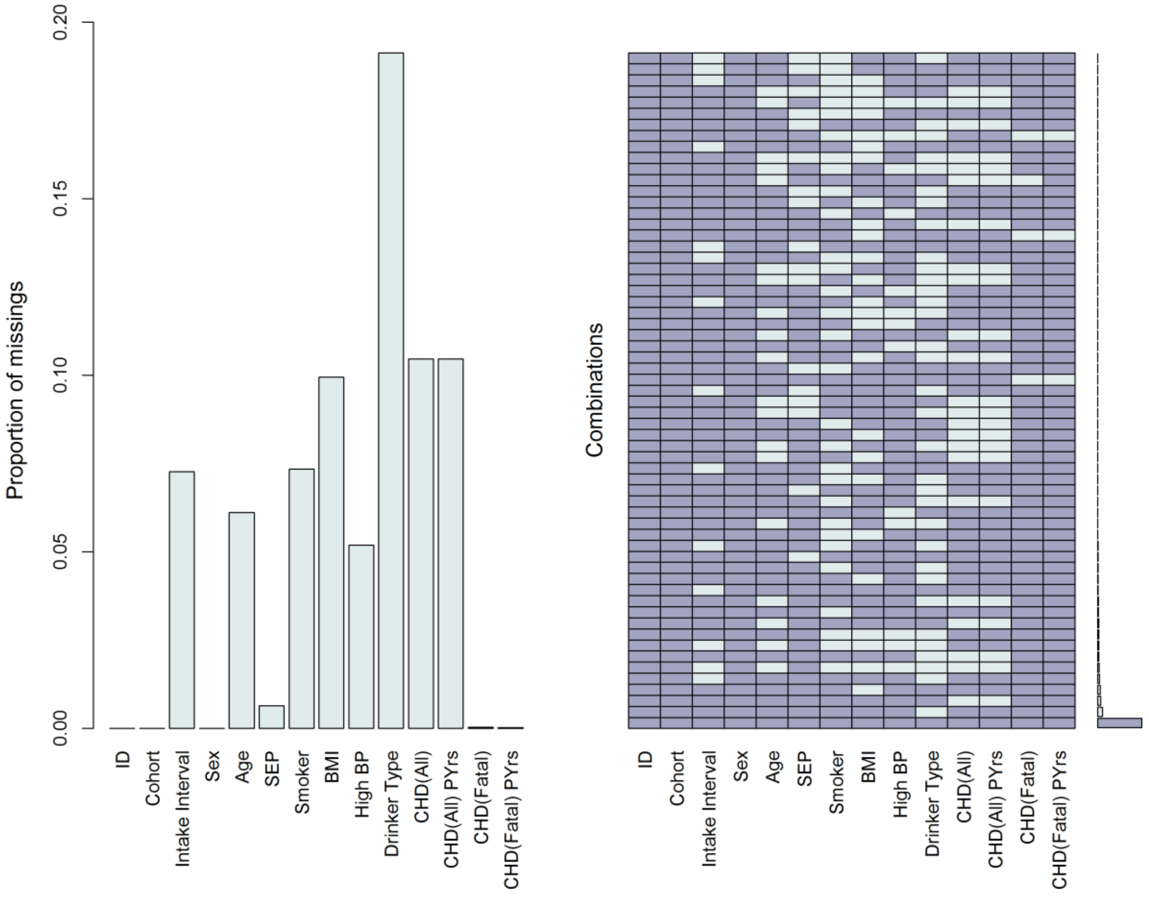

S5. Hierarchical Cox Regression Modelling Output

S5a. Incident CHD (Fatal & Non-Fatal) Modelling

Fixed Effects Table for Overall Sample: Incident CHD with Drinker Typology based on Single Intake Measure

Model	Covariate	log(HR)	SE	t	df	p	Lower 95%CI	Upper 95%CI
Partially Adjusted for Confounding	Non-drinker	0.26	0.06	4.07	8383.91	**<0.001**	0.13	0.38
	Heavy Drinker	-0.01	0.07	-0.20	8436.36	0.845	-0.15	0.13
	Intake Interval	-0.03	0.02	-1.65	206273.14	0.100	-0.07	0.01
	Age	0.07	0.00	19.16	354864.27	**<0.001**	0.07	0.08
	Sex: Female	-0.72	0.06	-12.70	785227.10	**<0.001**	-0.83	-0.61
Maximally Adjusted for Confounding	Non-drinker	0.23	0.06	3.66	7864.10	**<0.001**	0.11	0.36
	Heavy Drinker	-0.05	0.07	-0.76	8382.75	0.448	-0.20	0.09
	Intake Interval	-0.03	0.02	-1.57	204797.11	0.116	-0.07	0.01
	Age	0.08	0.00	19.16	304071.07	**<0.001**	0.07	0.08
	Sex: Female	-0.73	0.06	-12.60	781329.63	**<0.001**	-0.84	-0.62
	SEP: High	-0.06	0.05	-1.18	528975.70	0.239	-0.17	0.04
	SEP: Low	0.11	0.07	1.54	490117.85	0.125	-0.03	0.26
	Smoker: Current	0.53	0.08	6.63	30822.51	**<0.001**	0.37	0.68
	Smoker: Former	0.15	0.06	2.67	28620.06	**0.008**	0.04	0.26
Maximally Adjusted for Confounding and Mediation	Non-drinker	0.20	0.06	3.10	7022.18	**0.002**	0.07	0.32
	Heavy Drinker	-0.08	0.07	-1.16	8111.20	0.246	-0.23	0.06
	Intake Interval	-0.03	0.02	-1.40	198568.34	0.161	-0.07	0.01
	Age	0.07	0.00	18.11	250908.03	**<0.001**	0.06	0.08
	Sex: Female	-0.75	0.06	-12.98	711817.09	**<0.001**	-0.87	-0.64
	SEP: High	-0.06	0.05	-1.03	504039.06	0.303	-0.16	0.05
	SEP: Low	0.09	0.07	1.26	492218.60	0.209	-0.05	0.24
	Smoker: Current	0.56	0.08	6.98	31090.32	**<0.001**	0.40	0.71
	Smoker: Former	0.13	0.06	2.23	26625.41	**0.026**	0.02	0.23
	BMI	0.03	0.01	4.87	4463.71	**<0.001**	0.02	0.05
	High BP	0.47	0.05	8.62	25838.36	**<0.001**	0.36	0.57

Note: Significant values are highlighted in bold. Acronyms: SEP=Socioeconomic position; BMI=Body mass index; BP=Blood pressure. Reference categories: Drinker type=Moderate drinker; Sex=Male; SEP=Intermediate; Smoker: Non-smoker; High BP: Not reported.

Fixed Effects Table for Overall Sample: Incident CHD with Longitudinal Intake Typology

Model	Covariate	log(HR)	SE	t	df	p	Lower 95%CI	Upper 95%CI
Partially Adjusted for Confounding	Consistent Non-drinker	0.41	0.10	4.23	4236.16	**<0.001**	0.22	0.60
	Former Drinker	0.30	0.08	3.96	4730.07	**<0.001**	0.15	0.45
	Inconsistently Moderate Drinker	0.19	0.07	2.55	4043.69	**0.011**	0.04	0.34
	Consistently Heavy Drinker	0.09	0.11	0.81	4988.46	0.416	-0.12	0.30
	Inconsistently Heavy Drinker	0.15	0.10	1.42	5281.61	0.157	-0.06	0.35
	Intake Interval	-0.03	0.02	-1.69	177621.55	0.091	-0.07	0.01
	Age	0.07	0.00	19.28	355990.05	**<0.001**	0.07	0.08
	Sex: Female	-0.73	0.06	-12.79	631298.10	**<0.001**	-0.84	-0.62
Maximally Adjusted for Confounding	Consistent Non-drinker	0.38	0.10	3.91	4106.39	**<0.001**	0.19	0.57
	Former Drinker	0.27	0.08	3.49	4502.69	**<0.001**	0.12	0.42
	Inconsistently Moderate Drinker	0.17	0.08	2.23	3951.89	**0.026**	0.02	0.31
	Consistently Heavy Drinker	0.03	0.11	0.24	5074.57	0.811	-0.19	0.24
	Inconsistently Heavy Drinker	0.10	0.10	0.93	5705.02	0.352	-0.11	0.30
	Intake Interval	-0.03	0.02	-1.61	178500.19	0.107	-0.07	0.01
	Age	0.08	0.00	19.29	321678.95	**<0.001**	0.07	0.08
	Sex: Female	-0.74	0.06	-12.68	716030.76	**<0.001**	-0.85	-0.62
	SEP: High	-0.06	0.05	-1.13	381623.70	0.260	-0.17	0.04
	SEP: Low	0.11	0.07	1.49	578719.96	0.136	-0.04	0.26
	Smoker: Current	0.51	0.08	6.39	23221.78	**<0.001**	0.35	0.67
	Smoker: Former	0.15	0.06	2.58	16982.01	**0.010**	0.03	0.26
Maximally Adjusted for Confounding and Mediation	Consistent Non-drinker	0.36	0.10	3.66	4047.01	**<0.001**	0.17	0.55
	Former Drinker	0.22	0.08	2.88	4166.95	**0.004**	0.07	0.38
	Inconsistently Moderate Drinker	0.14	0.08	1.92	3669.78	0.055	0.00	0.29
	Consistently Heavy Drinker	-0.02	0.11	-0.19	4477.91	0.853	-0.24	0.19
	Inconsistently Heavy Drinker	0.06	0.10	0.56	5834.85	0.577	-0.15	0.26
	Intake Interval	-0.03	0.02	-1.44	167549.97	0.151	-0.07	0.01
	Age	0.07	0.00	18.22	304352.95	**<0.001**	0.06	0.08
	Sex: Female	-0.76	0.06	-13.07	678086.37	**<0.001**	-0.88	-0.65
	SEP: High	-0.05	0.05	-0.99	382236.91	0.322	-0.16	0.05
	SEP: Low	0.09	0.07	1.22	503067.27	0.224	-0.06	0.24
	Smoker: Current	0.54	0.08	6.77	22986.46	**<0.001**	0.39	0.70
	Smoker: Former	0.12	0.06	2.19	14952.86	**0.029**	0.01	0.24
	BMI	0.03	0.01	4.76	4914.86	**<0.001**	0.02	0.05
	High BP	0.47	0.05	8.79	66040.65	**<0.001**	0.37	0.58

Note: Significant values are highlighted in bold. Acronyms: SEP=Socioeconomic position; BMI=Body mass index; BP=Blood pressure. Reference categories: Drinker type=Consistently moderate drinker; Sex=Male; SEP=Intermediate; Smoker: Non-smoker; High BP: Not reported.

S5b. CHD Mortality Modelling

Fixed Effects Table for Overall Sample: Fatal CHD with Drinker Typology based on Single Intake Measure

Model	Covariate	log(HR)	SE	t	df	p	Lower 95%CI	Upper 95%CI
Partially Adjusted for Confounding	Non-drinker	0.45	0.14	3.14	3563.60	**0.002**	0.17	0.74
	Heavy Drinker	0.02	0.17	0.11	2533.58	0.915	-0.32	0.36
	Intake Interval	0.03	0.05	0.53	13650.23	0.596	-0.08	0.13
	Age	0.12	0.01	10.62	5638.26	**<0.001**	0.10	0.15
	Sex: Female	-0.86	0.13	-6.44	553255.23	**<0.001**	-1.12	-0.60
Maximally Adjusted for Confounding	Non-drinker	0.37	0.15	2.50	3242.23	**0.012**	0.08	0.66
	Heavy Drinker	-0.06	0.18	-0.37	2524.53	0.714	-0.41	0.28
	Intake Interval	0.02	0.05	0.46	14084.27	0.644	-0.08	0.13
	Age	0.13	0.01	10.91	5890.82	**<0.001**	0.10	0.15
	Sex: Female	-0.93	0.14	-6.84	575621.48	**<0.001**	-1.20	-0.66
	SEP: High	-0.20	0.13	-1.56	148522.59	0.118	-0.46	0.05
	SEP: Low	0.35	0.15	2.34	233161.08	**0.019**	0.06	0.65
	Smoker: Current	0.98	0.16	6.25	9125.37	**<0.001**	0.68	1.29
	Smoker: Former	0.09	0.15	0.59	7982.69	0.558	-0.20	0.37
Maximally Adjusted for Confounding and Mediation	Non-drinker	0.32	0.15	2.17	3287.87	**0.030**	0.03	0.61
	Heavy Drinker	-0.10	0.18	-0.57	2429.73	0.566	-0.45	0.24
	Intake Interval	0.02	0.05	0.45	11846.62	0.652	-0.08	0.13
	Age	0.12	0.01	10.58	6328.17	**<0.001**	0.10	0.15
	Sex: Female	-0.95	0.14	-6.95	392429.20	**<0.001**	-1.22	-0.68
	SEP: High	-0.19	0.13	-1.43	145223.22	0.151	-0.44	0.07
	SEP: Low	0.32	0.15	2.14	245944.42	**0.032**	0.03	0.62
	Smoker: Current	1.02	0.16	6.47	8867.24	**<0.001**	0.71	1.33
	Smoker: Former	0.05	0.15	0.35	7753.52	0.726	-0.24	0.34
	BMI	0.06	0.01	4.24	5335.63	**<0.001**	0.03	0.08
	High BP	0.51	0.13	4.01	14203.90	**<0.001**	0.26	0.76

Note: Significant values are highlighted in bold. Acronyms: SEP=Socioeconomic position; BMI=Body mass index; BP=Blood pressure. Reference categories: Drinker type=Moderate drinker; Sex=Male; SEP=Intermediate; Smoker: Non-smoker; High BP: Not reported.

Hazard Ratios and 95% Confidence Intervals for the Association of Drinker Type (Single Intake Measurement) with Fatal CHD using Maximal Adjustment for Confounding

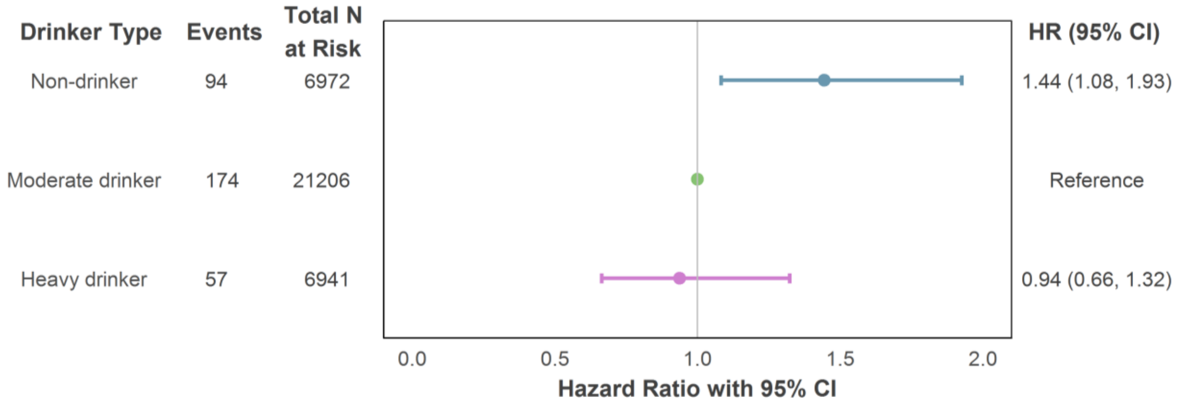

Note: Adjustment variables = age, sex (reference category: male), socioeconomic position (reference category: intermediate), smoker status (reference category: non-smoker), and intake assessment interval.

S5d. Fixed Effects Table for Overall Sample: Fatal CHD with Long-Term Intake Typology

Model	Covariate	log(HR)	SE	t	df	p	Lower 95%CI	Upper 95%CI
Partially Adjusted for Confounding	Consistent Non-drinker	0.54	0.23	2.38	1447.31	**0.017**	0.10	0.99
	Former Drinker	0.55	0.18	3.00	2132.17	**0.003**	0.19	0.91
	Inconsistently Moderate Drinker	0.09	0.19	0.46	1521.38	0.645	-0.29	0.46
	Consistently Heavy Drinker	0.14	0.27	0.53	1550.62	0.593	-0.38	0.66
	Inconsistently Heavy Drinker	0.43	0.22	1.92	1882.58	0.055	-0.01	0.86
	Intake Interval	0.02	0.05	0.45	17876.81	0.654	-0.08	0.13
	Age	0.12	0.01	10.68	5950.09	**<0.001**	0.10	0.15
	Sex: Female	-0.85	0.14	-6.29	145143.70	**<0.001**	-1.12	-0.59
Maximally Adjusted for Confounding	Consistent Non-drinker	0.42	0.23	1.82	1435.85	0.069	-0.03	0.87
	Former Drinker	0.43	0.19	2.33	2010.33	**0.020**	0.07	0.80
	Inconsistently Moderate Drinker	0.04	0.19	0.22	1520.88	0.825	-0.33	0.42
	Consistently Heavy Drinker	0.01	0.27	0.03	1537.51	0.978	-0.52	0.53
	Inconsistently Heavy Drinker	0.30	0.22	1.36	1908.82	0.175	-0.14	0.74
	Intake Interval	0.02	0.05	0.39	19536.04	0.694	-0.08	0.12
	Age	0.13	0.01	10.94	5873.84	**<0.001**	0.10	0.15
	Sex: Female	-0.92	0.14	-6.71	213139.02	**<0.001**	-1.19	-0.65
	SEP: High	-0.20	0.13	-1.55	169169.83	0.122	-0.45	0.05
	SEP: Low	0.36	0.15	2.34	229393.65	**0.019**	0.06	0.65
	Smoker: Current	0.95	0.16	6.08	14507.91	**<0.001**	0.64	1.25
	Smoker: Former	0.07	0.15	0.46	6710.42	0.643	-0.22	0.36
Maximally Adjusted for Confounding and Mediation	Consistent Non-drinker	0.37	0.23	1.58	1405.73	0.114	-0.09	0.82
	Former Drinker	0.37	0.19	1.96	1983.60	0.050	0.00	0.73
	Inconsistently Moderate Drinker	0.00	0.19	0.01	1498.08	0.992	-0.38	0.38
	Consistently Heavy Drinker	-0.05	0.27	-0.18	1567.16	0.856	-0.57	0.48
	Inconsistently Heavy Drinker	0.24	0.23	1.09	1888.16	0.277	-0.20	0.69
	Intake Interval	0.02	0.05	0.40	16449.43	0.692	-0.08	0.12
	Age	0.12	0.01	10.59	5994.47	**<0.001**	0.10	0.15
	Sex: Female	-0.94	0.14	-6.83	198370.99	**<0.001**	-1.21	-0.67
	SEP: High	-0.19	0.13	-1.43	154586.79	0.152	-0.44	0.07
	SEP: Low	0.33	0.15	2.15	201899.15	**0.032**	0.03	0.62
	Smoker: Current	0.99	0.16	6.35	16189.46	**<0.001**	0.68	1.29
	Smoker: Former	0.04	0.15	0.25	6743.61	0.802	-0.25	0.33
	BMI	0.06	0.01	4.26	5394.55	**<0.001**	0.03	0.08
	High BP	0.50	0.13	3.98	20239.66	**<0.001**	0.26	0.75

Note: Significant values are highlighted in bold. Acronyms: SEP=Socioeconomic position; BMI=Body mass index; BP=Blood pressure. Reference categories: Drinker type=Consistently moderate drinker; Sex=Male; SEP=Intermediate; Smoker: Non-smoker; High BP: Not reported.

Hazard Ratios and 95% Confidence Intervals for the Association of Drinker Type (Longitudinal Intake Measurement) with Fatal CHD using Maximal Adjustment for Confounding

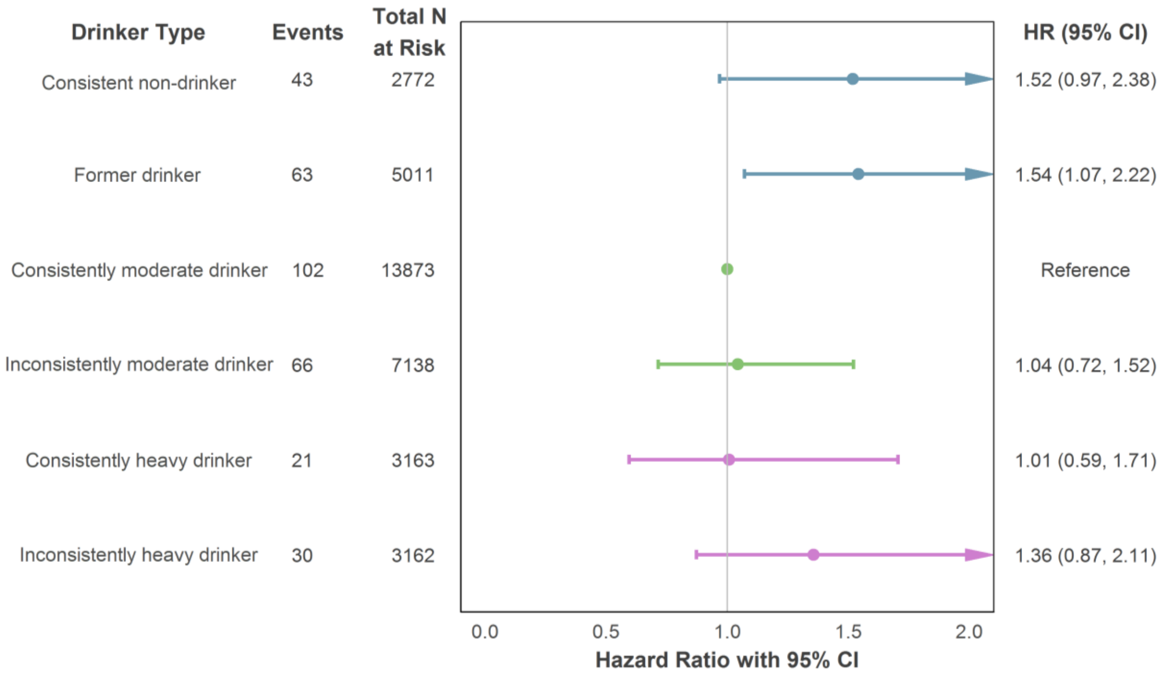

Note: Arrows on confidence interval bars signify where interval extends beyond the limits of the x-axis. The exact limits are presented in the 'HR (95%CI)' column. Adjustment variables = age, sex (reference category: male), socioeconomic position (reference category: intermediate), smoker status (reference category: non-smoker), and intake assessment interval.

S6. Age-Stratified Results: Longitudinal Intake Typology

S6a. Incident CHD (Fatal & Non-Fatal) Modelling

Fixed Effects Table: Participants Aged 55 or Younger (Incident CHD)

Model	Covariate	log(HR)	SE	t	df	p	Lower 95%CI	Upper 95%CI
Partially Adjusted for Confounding	Consistent Non-drinker	0.72	0.21	3.38	7517.02	**0.001**	0.30	1.14
	Former Drinker	0.53	0.20	2.71	14045.60	**0.007**	0.15	0.92
	Inconsistently Moderate Drinker	0.06	0.16	0.37	10619.85	0.709	-0.25	0.37
	Consistently Heavy Drinker	0.05	0.22	0.24	6922.69	0.814	-0.37	0.48
	Inconsistently Heavy Drinker	0.23	0.21	1.12	6229.96	0.265	-0.18	0.64
	Intake Interval	-0.01	0.07	-0.07	869631.74	0.942	-0.15	0.14
	Age	0.10	0.03	3.78	995536.57	**<0.001**	0.05	0.15
	Sex: Female	-0.95	0.15	-6.21	794252.17	**<0.001**	-1.24	-0.65
Maximally Adjusted for Confounding	Consistent Non-drinker	0.68	0.22	3.12	7036.07	**0.002**	0.25	1.10
	Former Drinker	0.47	0.20	2.38	13773.69	**0.018**	0.08	0.86
	Inconsistently Moderate Drinker	0.03	0.16	0.20	10458.93	0.844	-0.28	0.35
	Consistently Heavy Drinker	-0.03	0.22	-0.13	6377.97	0.899	-0.46	0.40
	Inconsistently Heavy Drinker	0.20	0.21	0.93	5902.57	0.351	-0.21	0.60
	Intake Interval	-0.01	0.07	-0.18	882880.93	0.860	-0.16	0.13
	Age	0.10	0.03	3.76	992707.96	**<0.001**	0.05	0.15
	Sex: Female	-1.00	0.16	-6.44	891235.07	**<0.001**	-1.30	-0.70
	SEP: High	-0.09	0.12	-0.72	952050.99	0.470	-0.32	0.15
	SEP: Low	0.18	0.19	0.91	873013.22	0.362	-0.20	0.56
	Smoker: Current	0.50	0.16	3.15	47562.24	**0.002**	0.19	0.81
	Smoker: Former	0.07	0.14	0.52	24375.29	0.601	-0.19	0.34
Maximally Adjusted for Confounding and Mediation	Consistent Non-drinker	0.64	0.22	2.94	7058.55	**0.003**	0.21	1.07
	Former Drinker	0.44	0.20	2.22	13600.00	**0.026**	0.05	0.83
	Inconsistently Moderate Drinker	0.01	0.16	0.05	10163.33	0.956	-0.31	0.32
	Consistently Heavy Drinker	-0.07	0.22	-0.34	6279.81	0.737	-0.50	0.36
	Inconsistently Heavy Drinker	0.15	0.21	0.70	5543.08	0.485	-0.26	0.56
	Intake Interval	-0.03	0.07	-0.37	754674.61	0.714	-0.17	0.12
	Age	0.09	0.03	3.44	943224.45	**0.001**	0.04	0.14
	Sex: Female	-1.01	0.16	-6.47	820117.66	**<0.001**	-1.32	-0.71
	SEP: High	-0.06	0.12	-0.50	894509.01	0.616	-0.30	0.18
	SEP: Low	0.14	0.19	0.71	644066.78	0.478	-0.24	0.52
	Smoker: Current	0.52	0.16	3.27	50298.51	**0.001**	0.21	0.83
	Smoker: Former	0.02	0.14	0.15	23889.16	0.881	-0.25	0.29
	BMI	0.05	0.01	3.15	3112.31	**0.002**	0.02	0.07
	High BP	0.46	0.14	3.34	17301.94	**0.001**	0.19	0.74

Note: Significant values are highlighted in bold. Acronyms: SEP=Socioeconomic position; BMI=Body mass index; BP=Blood pressure. Reference categories: Drinker type=Consistently moderate drinker; Sex=Male; SEP=Intermediate; Smoker: Non-smoker; High BP: Not reported.

Fixed Effects Table: Participants Aged above 55 (Incident CHD)

Model	Covariate	log(HR)	SE	t	df	p	Lower 95%CI	Upper 95%CI
Partially Adjusted for Confounding	Consistent Non-drinker	0.34	0.11	3.10	4648.74	**0.002**	0.13	0.56
	Former Drinker	0.27	0.09	3.14	3285.93	**0.002**	0.10	0.43
	Inconsistently Moderate Drinker	0.25	0.08	2.95	6891.20	**0.003**	0.08	0.41
	Consistently Heavy Drinker	0.13	0.12	1.02	9155.94	0.308	-0.12	0.37
	Inconsistently Heavy Drinker	0.13	0.12	1.07	6819.89	0.285	-0.11	0.37
	Intake Interval	-0.04	0.02	-1.91	553428.39	0.057	-0.08	0.00
	Age	0.07	0.00	14.29	989807.26	**<0.001**	0.06	0.08
	Sex: Female	-0.69	0.06	-11.02	629819.99	**<0.001**	-0.81	-0.57
Maximally Adjusted for Confounding	Consistent Non-drinker	0.32	0.11	2.88	4553.60	**0.004**	0.10	0.54
	Former Drinker	0.24	0.09	2.81	3240.83	**0.005**	0.07	0.41
	Inconsistently Moderate Drinker	0.23	0.08	2.69	6863.57	**0.007**	0.06	0.39
	Consistently Heavy Drinker	0.07	0.13	0.54	8659.65	0.590	-0.18	0.31
	Inconsistently Heavy Drinker	0.07	0.12	0.62	6679.28	0.539	-0.16	0.31
	Intake Interval	-0.04	0.02	-1.82	551775.21	0.069	-0.08	0.00
	Age	0.07	0.00	14.39	979852.71	**<0.001**	0.06	0.08
	Sex: Female	-0.68	0.06	-10.67	756373.90	**<0.001**	-0.81	-0.56
	SEP: High	-0.05	0.06	-0.75	426104.04	0.456	-0.16	0.07
	SEP: Low	0.08	0.08	0.91	499488.67	0.365	-0.09	0.24
	Smoker: Current	0.52	0.09	5.58	75333.87	**<0.001**	0.34	0.71
	Smoker: Former	0.16	0.06	2.54	73886.71	**0.011**	0.04	0.28
Maximally Adjusted for Confounding and Mediation	Consistent Non-drinker	0.30	0.11	2.67	4429.93	**0.008**	0.08	0.52
	Former Drinker	0.19	0.09	2.26	3280.55	**0.024**	0.03	0.36
	Inconsistently Moderate Drinker	0.20	0.08	2.43	6723.63	**0.015**	0.04	0.37
	Consistently Heavy Drinker	0.02	0.13	0.18	8366.50	0.854	-0.22	0.27
	Inconsistently Heavy Drinker	0.04	0.12	0.32	6779.11	0.751	-0.20	0.28
	Intake Interval	-0.03	0.02	-1.57	518873.15	0.116	-0.08	0.01
	Age	0.07	0.00	13.59	975491.13	**<0.001**	0.06	0.08
	Sex: Female	-0.71	0.06	-11.10	738744.22	**<0.001**	-0.84	-0.59
	SEP: High	-0.04	0.06	-0.71	419546.97	0.476	-0.16	0.08
	SEP: Low	0.06	0.08	0.67	505357.24	0.503	-0.11	0.22
	Smoker: Current	0.55	0.09	5.94	72988.61	**<0.001**	0.37	0.74
	Smoker: Former	0.14	0.06	2.28	74019.76	**0.023**	0.02	0.26

	BMI	0.03	0.01	3.73	12147.67	**<0.001**	0.01	0.04
	High BP	0.47	0.06	8.06	183650.76	**<0.001**	0.36	0.59

Note: Significant values are highlighted in bold. Acronyms: SEP=Socioeconomic position; BMI=Body mass index; BP=Blood pressure. Reference categories: Drinker type=Consistently moderate drinker; Sex=Male; SEP=Intermediate; Smoker: Non-smoker; High BP: Not reported.

S6b. CHD Mortality Modelling

Amongst participants over the 55 years age, former drinkers showed significantly greater risk than the moderate drinkers after adjustment for demographic and lifestyle characteristics (HR=1.48, 95%CI=1.0-2.18). A comparable finding was observed in the younger age group but the error bars in this group's estimates were so wide as to warrant caution in their interpretation, likely due to the low number of fatal CHD events amongst this group (HR=2.41, 95%CI=0.87-6.67).

Fixed Effects Table: Participants **Aged 55 or Younger** (Fatal CHD)

Model	Covariate	log(HR)	SE	t	df	p	Lower 95%CI	Upper 95%CI
Partially Adjusted for Confounding	Consistent Non-drinker	0.98	0.60	1.65	3344.89	0.100	-0.19	2.15
	Former Drinker	1.04	0.51	2.03	3750.02	**0.043**	0.03	2.05
	Inconsistently Moderate Drinker	0.02	0.51	0.04	5262.43	0.969	-0.97	1.01
	Consistently Heavy Drinker	0.64	0.54	1.18	4321.60	0.237	-0.42	1.71
	Inconsistently Heavy Drinker	0.93	0.49	1.90	5041.90	0.058	-0.03	1.89
	Intake Interval	-0.06	0.10	-0.63	996331.98	0.526	-0.26	0.13
	Age	-0.02	0.06	-0.31	993316.23	0.757	-0.14	0.10
	Sex: Female	-1.42	0.43	-3.30	455642.59	**0.001**	-2.26	-0.58
Maximally Adjusted for Confounding	Consistent Non-drinker	0.85	0.60	1.41	3212.09	0.158	-0.33	2.04
	Former Drinker	0.88	0.52	1.70	3643.58	0.090	-0.14	1.90
	Inconsistently Moderate Drinker	-0.04	0.51	-0.08	5008.47	0.936	-1.04	0.96
	Consistently Heavy Drinker	0.41	0.55	0.74	4082.43	0.457	-0.67	1.50
	Inconsistently Heavy Drinker	0.82	0.49	1.65	4796.96	0.098	-0.15	1.79
	Intake Interval	-0.10	0.11	-0.86	983695.34	0.389	-0.32	0.12
	Age	0.02	0.06	0.32	908439.00	0.751	-0.11	0.15
	Sex: Female	-1.47	0.43	-3.41	561672.26	**0.001**	-2.31	-0.62
	SEP: High	-0.39	0.32	-1.20	813687.05	0.229	-1.03	0.25
	SEP: Low	-0.23	0.50	-0.46	895556.34	0.647	-1.20	0.75
	Smoker: Current	1.20	0.35	3.39	23693.39	**0.001**	0.50	1.89
	Smoker: Former	-0.38	0.47	-0.82	9207.78	0.410	-1.29	0.53
Maximally Adjusted for	Consistent Non-drinker	0.79	0.61	1.30	3204.89	0.192	-0.40	1.98
	Former Drinker	0.83	0.52	1.59	3536.69	0.113	-0.20	1.85

Confounding and Mediation	Inconsistently Moderate Drinker	-0.08	0.51	-0.15	4900.23	0.882	-1.08	0.92
	Consistently Heavy Drinker	0.36	0.56	0.64	3924.82	0.522	-0.73	1.44
	Inconsistently Heavy Drinker	0.75	0.50	1.51	4847.80	0.131	-0.22	1.72
	Intake Interval	-0.12	0.12	-1.04	796437.18	0.300	-0.35	0.11
	Age	0.02	0.07	0.28	802308.30	0.780	-0.11	0.15
	Sex: Female	-1.48	0.44	-3.40	422104.08	**0.001**	-2.34	-0.63
	SEP: High	-0.38	0.32	-1.18	814062.89	0.239	-1.02	0.25
	SEP: Low	-0.31	0.51	-0.61	629570.60	0.544	-1.30	0.68
	Smoker: Current	1.22	0.35	3.44	23875.67	**0.001**	0.52	1.91
	Smoker: Former	-0.45	0.47	-0.97	8651.52	0.331	-1.37	0.46
	BMI	0.08	0.03	2.38	2781.17	**0.017**	0.01	0.15
	High BP	0.22	0.38	0.58	13725.19	0.560	-0.53	0.98

Note: Significant values are highlighted in bold. Acronyms: SEP=Socioeconomic position; BMI=Body mass index; BP=Blood pressure. Reference categories: Drinker type=Consistently moderate drinker; Sex=Male; SEP=Intermediate; Smoker: Non-smoker; High BP: Not reported.

Fixed Effects Table: Participants **Aged above 55** (Fatal CHD)

Model	Covariate	log(HR)	SE	t	df	p	Lower 95%CI	Upper 95%CI
Partially Adjusted for Confounding	Consistent Non-drinker	0.40	0.26	1.55	2699.29	0.122	-0.11	0.91
	Former Drinker	0.47	0.20	2.41	5910.23	**0.016**	0.09	0.86
	Inconsistently Moderate Drinker	0.08	0.21	0.38	4270.38	0.705	-0.34	0.50
	Consistently Heavy Drinker	-0.12	0.33	-0.37	2436.40	0.713	-0.78	0.53
	Inconsistently Heavy Drinker	0.23	0.28	0.82	1997.41	0.412	-0.32	0.79
	Intake Interval	-0.03	0.06	-0.50	121561.27	0.615	-0.15	0.09
	Age	0.14	0.01	9.38	988837.23	**<0.001**	0.11	0.17
	Sex: Female	-0.80	0.16	-5.11	516369.11	**<0.001**	-1.11	-0.50
Maximally Adjusted for Confounding	Consistent Non-drinker	0.32	0.26	1.24	2754.98	0.213	-0.19	0.83
	Former Drinker	0.39	0.20	1.98	5841.05	**0.048**	0.00	0.78
	Inconsistently Moderate Drinker	0.05	0.21	0.22	4197.73	0.823	-0.37	0.46
	Consistently Heavy Drinker	-0.24	0.34	-0.72	2421.29	0.471	-0.90	0.42
	Inconsistently Heavy Drinker	0.11	0.28	0.40	2067.86	0.688	-0.44	0.67
	Intake Interval	-0.03	0.06	-0.51	122559.53	0.611	-0.14	0.08
	Age	0.14	0.01	9.59	966969.62	**<0.001**	0.11	0.17
	Sex: Female	-0.85	0.16	-5.28	577201.24	**<0.001**	-1.16	-0.53
	SEP: High	-0.04	0.15	-0.29	472168.23	0.774	-0.34	0.25
	SEP: Low	0.40	0.18	2.17	372220.44	**0.030**	0.04	0.75
	Smoker: Current	0.92	0.19	4.92	55893.23	**<0.001**	0.55	1.29

	Smoker: Former	0.10	0.16	0.63	24423.34	0.527	-0.22	0.42
Maximally Adjusted for Confounding and Mediation	Consistent Non-drinker	0.28	0.26	1.06	2611.00	0.291	-0.24	0.79
	Former Drinker	0.31	0.20	1.57	5781.69	0.117	-0.08	0.71
	Inconsistently Moderate Drinker	-0.01	0.21	-0.03	4192.19	0.977	-0.42	0.41
	Consistently Heavy Drinker	-0.31	0.34	-0.92	2471.73	0.358	-0.97	0.35
	Inconsistently Heavy Drinker	0.05	0.29	0.17	2034.26	0.869	-0.51	0.61
	Intake Interval	-0.02	0.06	-0.31	112637.67	0.759	-0.13	0.10
	Age	0.14	0.01	9.29	932394.82	**<0.001**	0.11	0.17
	Sex: Female	-0.90	0.16	-5.59	480889.59	**<0.001**	-1.22	-0.59
	SEP: High	-0.03	0.15	-0.22	462944.45	0.823	-0.33	0.26
	SEP: Low	0.36	0.18	1.98	364765.91	**0.047**	0.00	0.72
	Smoker: Current	0.99	0.19	5.30	64928.73	**<0.001**	0.62	1.35
	Smoker: Former	0.07	0.16	0.40	25432.72	0.689	-0.25	0.39
	BMI	0.07	0.02	4.23	5464.74	**<0.001**	0.04	0.10
	High BP	0.63	0.14	4.51	74368.28	**<0.001**	0.36	0.91

Note: Significant values are highlighted in bold. Acronyms: SEP=Socioeconomic position; BMI=Body mass index; BP=Blood pressure. Reference categories: Drinker type=Consistently moderate drinker; Sex=Male; SEP=Intermediate; Smoker: Non-smoker; High BP: Not reported.

S7. Sex-Stratified Results: Longitudinal Intake Typology

S7a. Incident CHD (Fatal & Non-Fatal) *Modelling*

Fixed Effects Table: **Male** Participants (Incident CHD)

Model	Covariate	log(HR)	SE	t	df	p	Lower 95%CI	Upper 95%CI
Partially Adjusted for Confounding	Consistent Non-drinker	0.15	0.14	1.09	4152.46	0.277	-0.12	0.43
	Former Drinker	0.29	0.10	2.97	3746.13	**0.003**	0.10	0.48
	Inconsistently Moderate Drinker	0.17	0.09	1.97	3153.03	**0.049**	0.00	0.34
	Consistently Heavy Drinker	0.12	0.11	1.05	3605.15	0.295	-0.10	0.34
	Inconsistently Heavy Drinker	0.09	0.12	0.81	3771.82	0.417	-0.13	0.32
	Intake Interval	-0.01	0.02	-0.46	185474.45	0.646	-0.06	0.04
	Age	0.07	0.00	14.32	232926.25	**<0.001**	0.06	0.08
Maximally Adjusted for Confounding	Consistent Non-drinker	0.13	0.14	0.94	4251.20	0.346	-0.14	0.41
	Former Drinker	0.26	0.10	2.60	3705.25	**0.009**	0.06	0.45
	Inconsistently Moderate Drinker	0.15	0.09	1.70	3048.71	0.090	-0.02	0.32
	Consistently Heavy Drinker	0.05	0.12	0.42	3520.60	0.677	-0.18	0.27
	Inconsistently Heavy Drinker	0.04	0.12	0.32	3528.86	0.745	-0.19	0.27
	Intake Interval	-0.01	0.03	-0.41	185679.73	0.684	-0.06	0.04
	Age	0.07	0.00	14.34	198392.71	**<0.001**	0.06	0.08
	SEP: High	-0.06	0.06	-0.90	571534.36	0.367	-0.18	0.06
	SEP: Low	0.04	0.10	0.36	567858.41	0.715	-0.16	0.24
	Smoker: Current	0.53	0.09	5.65	30074.37	**<0.001**	0.35	0.72
	Smoker: Former	0.15	0.07	2.17	18405.38	**0.030**	0.01	0.28
Maximally Adjusted for Confounding and Mediation	Consistent Non-drinker	0.12	0.14	0.86	4119.43	0.392	-0.16	0.40
	Former Drinker	0.22	0.10	2.25	3572.42	**0.024**	0.03	0.42
	Inconsistently Moderate Drinker	0.12	0.09	1.37	2863.32	0.171	-0.05	0.29
	Consistently Heavy Drinker	0.00	0.12	0.00	3558.36	0.997	-0.23	0.23
	Inconsistently Heavy Drinker	-0.01	0.12	-0.11	3476.05	0.911	-0.24	0.22
	Intake Interval	-0.01	0.03	-0.44	182889.65	0.663	-0.06	0.04
	Age	0.07	0.00	13.74	155949.69	**<0.001**	0.06	0.08
	SEP: High	-0.05	0.06	-0.76	490527.15	0.447	-0.17	0.07
	SEP: Low	0.03	0.10	0.30	549528.30	0.763	-0.17	0.23
	Smoker: Current	0.56	0.09	5.91	27685.48	**<0.001**	0.37	0.74
	Smoker: Former	0.11	0.07	1.68	16900.62	0.094	-0.02	0.25
	BMI	0.04	0.01	4.78	3620.72	**<0.001**	0.03	0.06
	High BP	0.42	0.07	6.44	25521.00	**<0.001**	0.29	0.55

Note: Significant values are highlighted in bold. Acronyms: SEP=Socioeconomic position; BMI=Body mass index; BP=Blood pressure. Reference categories: Drinker type=Consistently moderate drinker; SEP=Intermediate; Smoker: Non-smoker; High BP: Not reported.

Fixed Effects Table: **Female** Participants (Incident CHD)

Model	Covariate	log(HR)	SE	t	df	p	Lower 95%CI	Upper 95%CI
Partially Adjusted for Confounding	Consistent Non-drinker	0.66	0.15	4.54	3469.09	**<0.001**	0.38	0.95
	Former Drinker	0.35	0.13	2.73	6803.12	**0.006**	0.10	0.60
	Inconsistently Moderate Drinker	0.25	0.15	1.66	6745.60	0.097	-0.05	0.55
	Consistently Heavy Drinker	-0.80	0.50	-1.59	7788.92	0.111	-1.78	0.18
	Inconsistently Heavy Drinker	0.26	0.25	1.05	4349.33	0.296	-0.23	0.74
	Intake Interval	-0.07	0.03	-1.97	353814.36	**0.048**	-0.13	0.00
	Age	0.09	0.01	12.82	206387.60	**<0.001**	0.08	0.10
Maximally Adjusted for Confounding	Consistent Non-drinker	0.65	0.15	4.35	3385.79	**<0.001**	0.36	0.94
	Former Drinker	0.32	0.13	2.50	6383.83	**0.012**	0.07	0.57
	Inconsistently Moderate Drinker	0.23	0.15	1.53	6737.32	0.126	-0.07	0.53
	Consistently Heavy Drinker	-0.82	0.50	-1.64	7740.19	0.101	-1.81	0.16
	Inconsistently Heavy Drinker	0.23	0.25	0.93	4308.33	0.353	-0.26	0.72
	Intake Interval	-0.06	0.03	-1.94	352075.72	0.053	-0.13	0.00
	Age	0.09	0.01	12.82	198859.17	**<0.001**	0.08	0.10
	SEP: High	-0.07	0.11	-0.61	256571.43	0.543	-0.29	0.15
	SEP: Low	0.14	0.12	1.24	342591.08	0.215	-0.08	0.37
	Smoker: Current	0.46	0.15	3.02	21713.42	**<0.001**	0.16	0.76
	Smoker: Former	0.19	0.10	1.86	18319.64	0.063	-0.01	0.40
Maximally Adjusted for Confounding and Mediation	Consistent Non-drinker	0.62	0.15	4.19	3608.40	**<0.001**	0.33	0.91
	Former Drinker	0.27	0.13	2.08	5627.04	**0.038**	0.02	0.52
	Inconsistently Moderate Drinker	0.22	0.15	1.42	6809.80	0.155	-0.08	0.52
	Consistently Heavy Drinker	-0.84	0.50	-1.68	7836.10	0.093	-1.83	0.14
	Inconsistently Heavy Drinker	0.24	0.25	0.98	4091.84	0.329	-0.25	0.73
	Intake Interval	-0.05	0.03	-1.58	339454.70	0.113	-0.12	0.01
	Age	0.08	0.01	11.89	180148.16	**<0.001**	0.07	0.10
	SEP: High	-0.06	0.11	-0.55	255763.84	0.585	-0.28	0.16
	SEP: Low	0.12	0.12	1.05	344293.37	0.292	-0.11	0.35
	Smoker: Current	0.50	0.15	3.28	21324.01	**0.001**	0.20	0.80
	Smoker: Former	0.17	0.11	1.64	17635.35	0.100	-0.03	0.38
	BMI	0.02	0.01	1.68	4188.43	0.093	0.00	0.04
	High BP	0.56	0.10	5.64	20983.30	**<0.001**	0.37	0.76

Note: Significant values are highlighted in bold. Acronyms: SEP=Socioeconomic position; BMI=Body mass index; BP=Blood pressure. Reference categories: Drinker type=Consistently moderate drinker; SEP=Intermediate; Smoker: Non-smoker; High BP: Not reported.

S7b. CHD Mortality Modelling

Male former drinkers again showed greater risk than the consistently moderate category (HR=1.63, 95%CI=1.06-2.53) following adjustment for age, sex and intake assessment interval, but this difference was no longer significant once smoking status and socioeconomic position were accounted for. Amongst the female participants, due to the low heavy drinker count, it was necessary to merge consistently and inconsistently heavy drinkers into a single category for the CHD mortality analysis, but this combined drinker group nonetheless did not differ statistically in risk from the consistently moderate reference group. Female consistent non-drinkers (HR=2.61, 95%CI=1.25-5.47) however did have significantly greater risk of fatal CHD than those who consistently drank moderately. Female former drinkers also demonstrated a significantly greater risk of fatal CHD (HR=2.12, 95%CI=1.06-4.22), an effect that was no longer significant after adjustment was made for clinical characteristics.

Fixed Effects Table: **Male** Participants (Fatal CHD)

Model	Covariate	log(HR)	SE	t	df	p	Lower 95%CI	Upper 95%CI
Partially Adjusted for Confounding	Consistent Non-drinker	0.16	0.32	0.51	2178.12	0.611	-0.47	0.80
	Former Drinker	0.49	0.22	2.21	1743.79	**0.027**	0.06	0.93
	Inconsistently Moderate Drinker	0.01	0.22	0.03	1388.19	0.977	-0.42	0.43
	Consistently Heavy Drinker	0.07	0.28	0.25	1439.46	0.804	-0.48	0.61
	Inconsistently Heavy Drinker	0.24	0.25	0.99	1888.32	0.322	-0.24	0.73
	Intake Interval	0.03	0.06	0.51	29120.69	0.612	-0.09	0.16
	Age	0.11	0.01	8.36	5330.37	**<0.001**	0.09	0.14
Maximally Adjusted for Confounding	Consistent Non-drinker	0.04	0.32	0.13	2205.13	0.894	-0.59	0.68
	Former Drinker	0.37	0.22	1.64	1696.70	0.102	-0.07	0.81
	Inconsistently Moderate Drinker	-0.04	0.22	-0.18	1417.18	0.857	-0.47	0.39
	Consistently Heavy Drinker	-0.08	0.28	-0.28	1406.22	0.782	-0.63	0.47
	Inconsistently Heavy Drinker	0.12	0.25	0.47	1877.83	0.636	-0.37	0.61
	Intake Interval	0.03	0.06	0.47	28323.57	0.641	-0.09	0.15
	Age	0.12	0.01	8.69	5783.95	**<0.001**	0.09	0.14
	SEP: High	-0.30	0.14	-2.06	210062.19	**0.040**	-0.58	-0.01
	SEP: Low	0.19	0.20	0.95	174686.50	0.344	-0.20	0.58
	Smoker: Current	0.94	0.18	5.15	21956.15	**<0.001**	0.58	1.29
	Smoker: Former	0.06	0.17	0.33	12340.30	0.739	-0.28	0.39
Maximally Adjusted for Confounding and Mediation	Consistent Non-drinker	0.00	0.33	0.00	2143.43	0.998	-0.64	0.64
	Former Drinker	0.33	0.22	1.46	1695.24	0.145	-0.11	0.77
	Inconsistently Moderate Drinker	-0.09	0.22	-0.40	1396.14	0.692	-0.52	0.34
	Consistently Heavy Drinker	-0.13	0.28	-0.47	1447.90	0.641	-0.68	0.42

Inconsistently Heavy Drinker	0.05	0.25	0.21	1934.94	0.833	-0.44	0.54
Intake Interval	0.02	0.06	0.39	22976.36	0.694	-0.10	0.15
Age	0.12	0.01	8.47	5866.02	**<0.001**	0.09	0.14
SEP: High	-0.28	0.14	-1.95	186806.07	0.051	-0.56	0.00
SEP: Low	0.18	0.20	0.92	169070.17	0.356	-0.21	0.57
Smoker: Current	0.96	0.18	5.30	21848.51	**<0.001**	0.61	1.32
Smoker: Former	0.02	0.17	0.12	12195.90	0.908	-0.31	0.35
BMI	0.06	0.02	3.22	4472.11	**0.001**	0.02	0.09
High BP	0.55	0.15	3.68	13588.55	**<0.001**	0.26	0.84

Note: Significant values are highlighted in bold. Acronyms: SEP=Socioeconomic position; BMI=Body mass index; BP=Blood pressure. Reference categories: Drinker type=Consistently moderate drinker; SEP=Intermediate; Smoker: Non-smoker; High BP: Not reported.

Fixed Effects Table: **Female** Participants (Fatal CHD)

Model	Covariate	log(HR)	SE	t	df	p	Lower 95%CI	Upper 95%CI
Partially Adjusted for Confounding	Consistent Non-drinker	1.05	0.37	2.83	1970.19	**0.005**	0.32	1.78
	Former Drinker	0.84	0.35	2.41	2994.07	**0.016**	0.16	1.53
	Inconsistently Moderate Drinker	0.37	0.44	0.85	1282.80	0.396	-0.49	1.23
	Consistently/Inconsistently Heavy Drinker	0.87	0.49	1.79	2270.15	0.074	-0.09	1.83
	Intake Interval	-0.01	0.08	-0.11	26926.32	0.913	-0.17	0.15
	Age	0.14	0.02	6.81	7549.67	**<0.001**	0.10	0.18
Maximally Adjusted for Confounding	Consistent Non-drinker	0.96	0.38	2.55	1865.43	**0.011**	0.22	1.70
	Former Drinker	0.75	0.35	2.12	2877.45	**0.034**	0.06	1.44
	Inconsistently Moderate Drinker	0.33	0.44	0.76	1298.50	0.448	-0.53	1.19
	Consistently/Inconsistently Heavy Drinker	0.79	0.49	1.59	2235.71	0.111	-0.18	1.75
	Intake Interval	-0.01	0.07	-0.13	36225.55	0.898	-0.15	0.13
	Age	0.14	0.02	7.23	8974.65	**<0.001**	0.10	0.18
	SEP: High	0.14	0.29	0.47	187259.92	0.639	-0.44	0.72
	SEP: Low	0.62	0.27	2.33	280611.60	**0.020**	0.10	1.15
	Smoker: Current	1.06	0.30	3.54	9088.78	**<0.001**	0.47	1.64
	Smoker: Former	0.19	0.29	0.65	5596.17	0.517	-0.38	0.75
Maximally Adjusted for Confounding and Mediation	Consistent Non-drinker	0.89	0.38	2.33	1835.72	**0.020**	0.14	1.63
	Former Drinker	0.65	0.35	1.83	2956.26	0.067	-0.05	1.34
	Inconsistently Moderate Drinker	0.31	0.44	0.70	1286.55	0.485	-0.55	1.16
	Consistently/Inconsistently Heavy Drinker	0.78	0.49	1.58	2149.33	0.114	-0.19	1.75
	Intake Interval	-0.02	0.07	-0.25	36548.04	0.803	-0.16	0.12
	Age	0.14	0.02	7.26	9948.67	**<0.001**	0.10	0.17
	SEP: High	0.16	0.29	0.53	193405.77	0.594	-0.42	0.73
	SEP: Low	0.58	0.27	2.17	301190.49	**0.030**	0.06	1.10
	Smoker: Current	1.14	0.30	3.80	8731.17	**<0.001**	0.55	1.72
	Smoker: Former	0.15	0.29	0.51	5435.40	0.609	-0.42	0.71
	BMI	0.06	0.02	2.76	3491.86	**0.006**	0.02	0.10
	High BP	0.33	0.25	1.33	10219.76	0.183	-0.15	0.81

Note: Consistently and inconsistently heavy drinkers are combined in the female subsample due to the low event count for these drinker types. Significant values are highlighted in bold. Acronyms: SEP=Socioeconomic position; BMI=Body mass index; BP=Blood pressure. Reference categories: Drinker type=Consistently moderate drinker; SEP=Intermediate; Smoker: Non-smoker; High BP: Not reported.

The present study attempted to maximise identification of heavy consumers in the female sample by unifying the consistently and inconsistently heavy drinker categories for the CHD mortality analysis in the sex-stratified analyses, but no significant effects were found. A similar absence of effect was

found in the pooled sample and for the male-only subsample when heavy drinker categories were merged in supplementary analyses (illustrated in the figures below).

Hazard Ratios and 95% Confidence Intervals for the Association of Drinker Type (Longitudinal Intake Measurement with Combined Consistently/Inconsistently Heavy Drinker Category) with Fatal CHD using Maximal Adjustment for Confounding

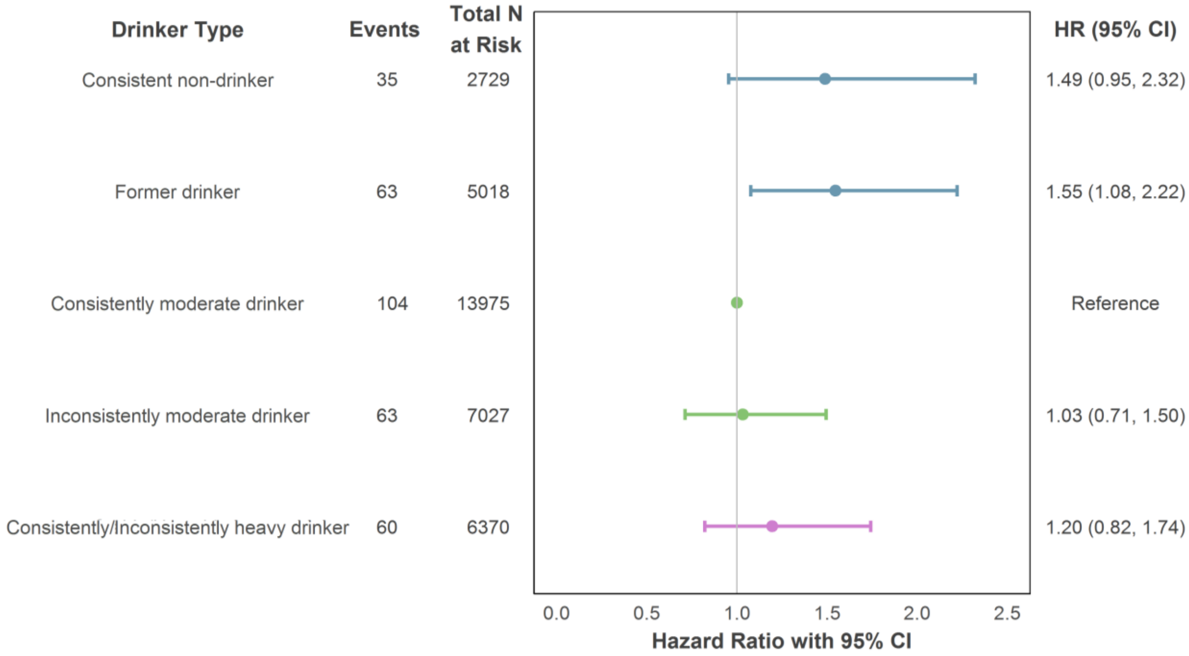

Note: Adjustment variables = age, sex (reference category: male), socioeconomic position (reference category: intermediate), smoker status (reference category: non-smoker), and intake assessment interval.

Hazard Ratios and 95% Confidence Intervals for the Association of Drinker Type (Longitudinal Intake Measurement with Combined Consistently/Inconsistently Heavy Drinker Category) with Fatal CHD using Maximal Adjustment for Confounding: Male Participants Only

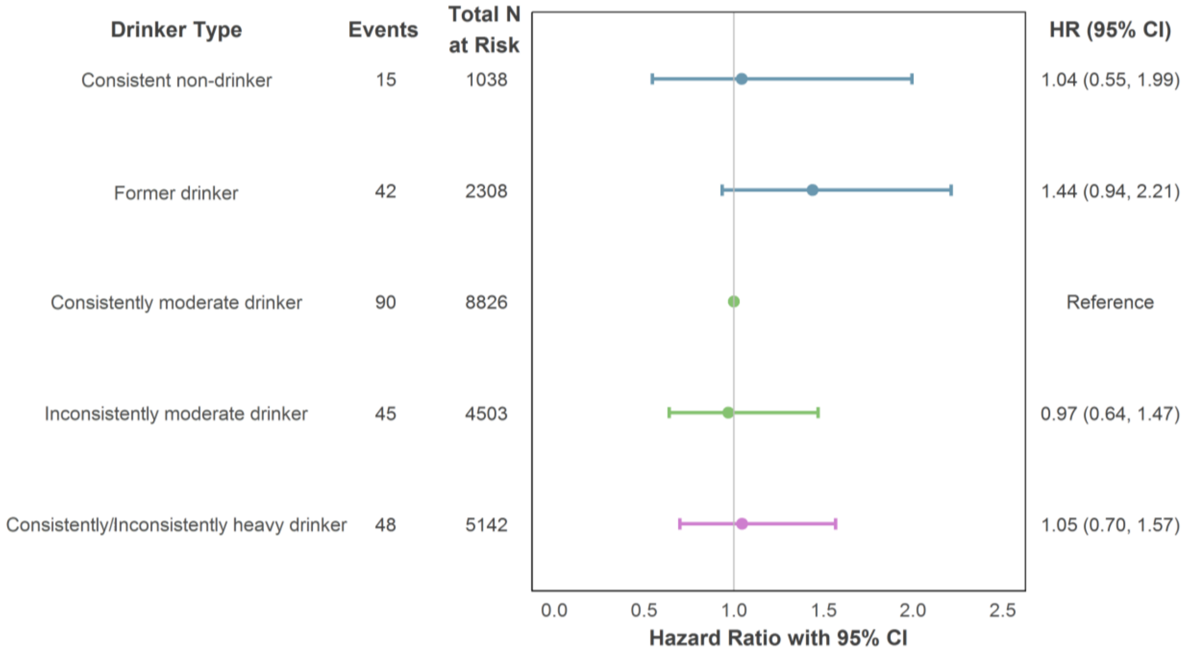

Note: Adjustment variables = age, socioeconomic position (reference category: intermediate), smoker status (reference category: non-smoker), and intake assessment interval.

S8. Fixed Effects Plots: UK Cohorts Only

Hazard Ratios and 95% Confidence Intervals for the Association of Drinker Type (Longitudinal Intake Measurement) with Incident (Fatal/Non-Fatal) CHD using Maximal Adjustment for Confounding: UK Cohort Only Sample

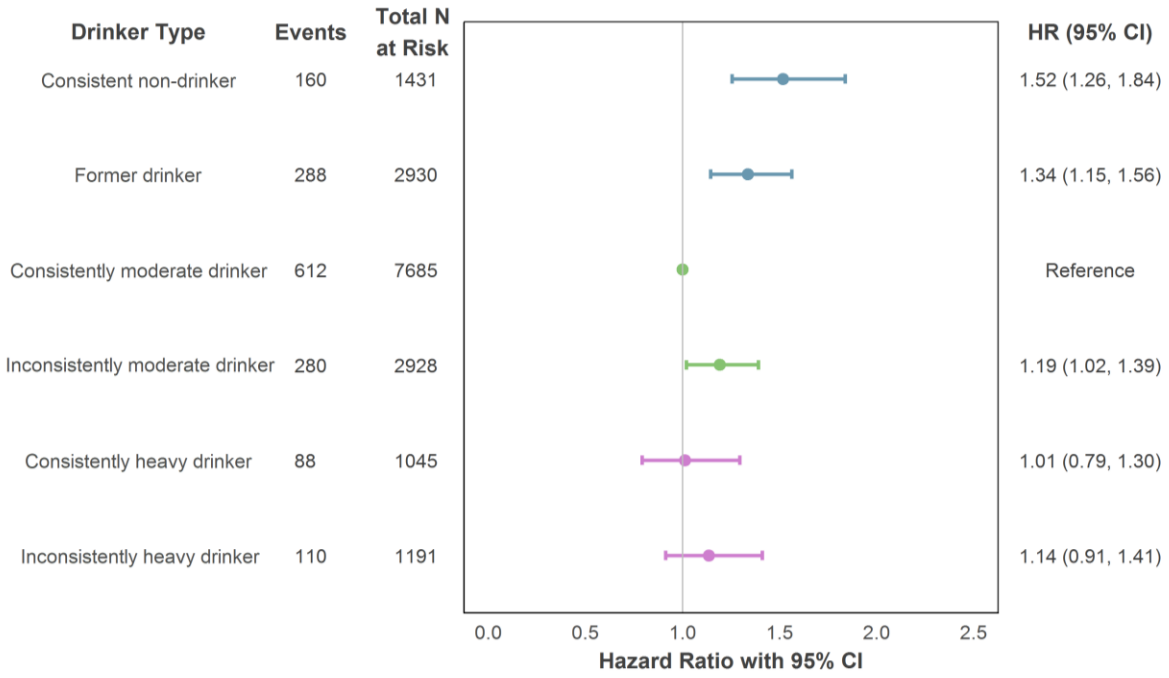

Note: Adjustment variables = age, sex (reference category: male), socioeconomic position (reference category: intermediate), smoker status (reference category: non-smoker), and intake assessment interval.

Hazard Ratios and 95% Confidence Intervals for the Association of Drinker Type (Longitudinal Intake Measurement) with Fatal CHD using Maximal Adjustment for Confounding: UK Cohort Only Sample

Drinker Type	Events	Total N at Risk	HR (95% CI)
Consistent non-drinker	35	1851	1.71 (1.08, 2.72)
Former drinker	59	3483	1.78 (1.20, 2.63)
Consistently moderate drinker	81	8802	Reference
Inconsistently moderate drinker	49	3950	1.11 (0.72, 1.70)
Consistently heavy drinker	12	1222	1.11 (0.57, 2.15)
Inconsistently heavy drinker	33	1564	1.56 (0.93, 2.61)

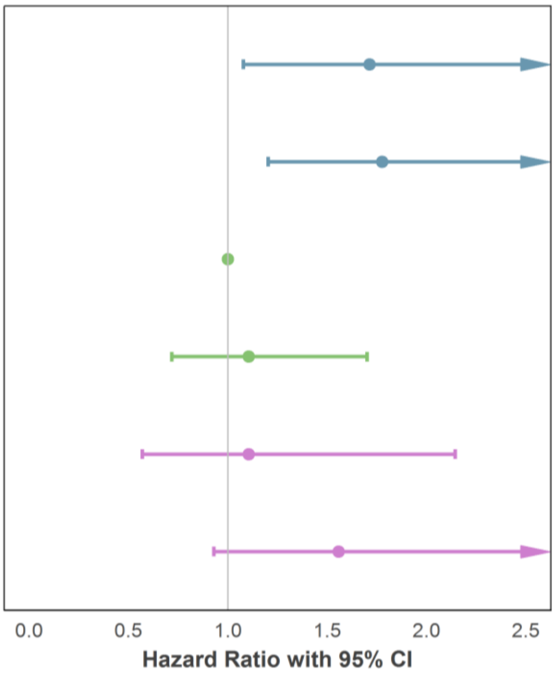

Note: Adjustment variables = age, sex (reference category: male), socioeconomic position (reference category: intermediate), smoker status (reference category: non-smoker), and intake assessment interval.

S9. Fixed Effects Plots: Complete Case Data

Hazard Ratios and 95% Confidence Intervals for the Association of Drinker Type (Longitudinal Intake Measurement) with Incident (Fatal/Non-Fatal) CHD using Maximal Adjustment for Confounding: Complete Case Data Only

Drinker Type	Events	Total N at Risk	HR (95% CI)
Consistent non-drinker	129	1932	1.49 (1.21, 1.82)
Former drinker	250	3799	1.32 (1.12, 1.54)
Consistently moderate drinker	560	10820	Reference
Inconsistently moderate drinker	264	5039	1.19 (1.02, 1.39)
Consistently heavy drinker	98	2473	1.06 (0.85, 1.33)
Inconsistently heavy drinker	107	2336	1.11 (0.90, 1.38)

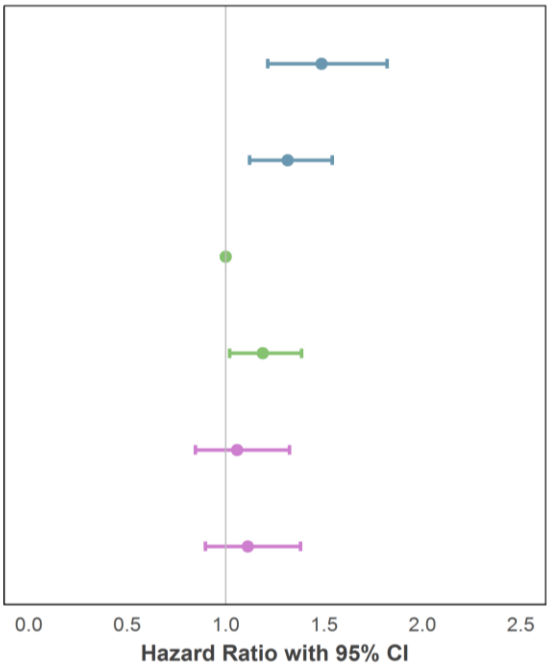

Note: Adjustment variables = age, sex (reference category: male), socioeconomic position (reference category: intermediate), smoker status (reference category: non-smoker), and intake assessment interval.

Hazard Ratios and 95% Confidence Intervals for the Association of Drinker Type (Longitudinal Intake Measurement) with Fatal CHD using Maximal Adjustment for Confounding: Complete Case Data Only

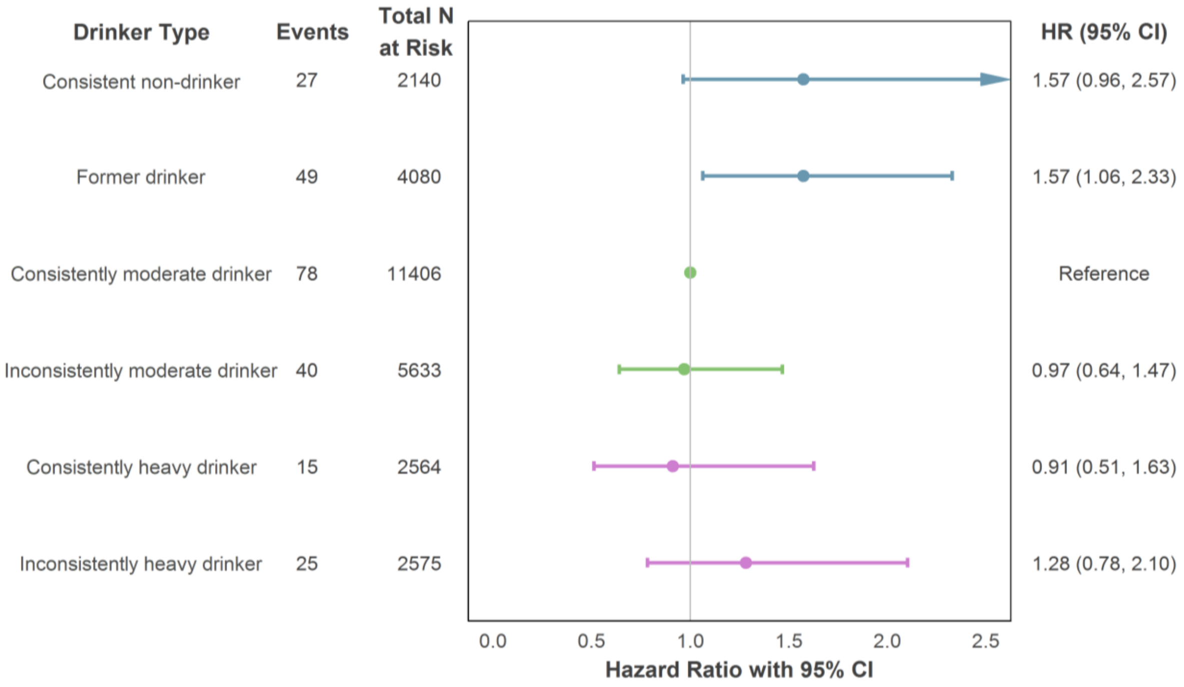

Note: Adjustment variables = age, sex (reference category: male), socioeconomic position (reference category: intermediate), smoker status (reference category: non-smoker), and intake assessment interval.

S10. Study Enrolment and Follow-up Procedures

The following sections outline the enrolment and follow-up assessment procedures for each of the cohorts from which the study participants were sampled. For each of the cohorts, time-to-event for fatal and non-fatal CHD was calculated for all participants as time (in years) between the date of the final alcohol assessment and either (i) the date of the CHD event, (ii) death from non-CHD causes, (iii) study dropout or (iv) last date of data linkage, whichever occurred first.

EPIC-Norfolk

Recruitment for this regional cohort occurred between 1993 and 1997. Potential participants were identified from general practitioner databases in Norfolk, United Kingdom. Of 77,630 invitations sent out, 25,633 were successfully recruited. Following the current study's inclusion criteria as outlined in the main manuscript, 7,462 participants were sampled for the present study. The alcohol intake data used for the longitudinal exposure variable were collected initially during 1993-1997, with a second assessment during 1997-2000 and a final third assessment during 2006-2011. Fatal and non-fatal coronary heart disease (CHD) diagnoses and time-to-event data were determined using linked administrative health records. The diagnoses were based on the International Classification of Disease (ICD) codes for ischaemic heart disease: 410-414 (ICD-9) I20-I25 (ICD-10). The mean follow-up time for incident CHD was 6.9 years.

GAZEL

Initial recruitment for this cohort commenced in 1989, targeting employees of a national utility company in France, with the recruited sample comprising 20,624 participants. Of these, 14,247 participants met the current study's inclusion criteria. Alcohol intake assessments for these participants were sampled from questionnaire data from 1992, 1997 and 2002. Data on non-fatal CHD events were first self-reported by participants, and then validated using company medical record data. These medical data were also used to determine the occurrence and time-to-event for fatal CHD events. CHD diagnoses were classified according to the ICD codes for ischaemic heart disease: 410-414 (ICD-9) or I20-I25 (ICD-10). The mean follow-up time for incident CHD was 14.8 years.

NSHD

This birth cohort is based on a socially-stratified sample recruited from all British born children born in one week in 1946. In 1982, the study had 3,322 participants and of these 2,979 were eligible for inclusion in the current study. Longitudinal alcohol intake patterns were determinable using

reported consumption levels in 1982, 1989 and 1999. In terms of CHD outcomes, non-fatal CHD events were identified for NSHD, but time-to-event from the end of the exposure period was not ascertainable so this study was omitted from analysis of the incident CHD endpoint. Survival time was determinable for fatal CHD events through hospital record linkage, and this study was consequently included in the CHD mortality analyses. CHD events were diagnosed using the ICD codes for ischaemic heart disease: 410-414 (ICD-9) or I20-I25 (ICD-10). The mean follow-up time for CHD mortality events was 14.5 years.

Twenty-07 1930's and Twenty-07 1950's

The West of Scotland Twenty-07 regional cohorts were established in 1986. One of the cohorts comprised 1,551 individuals born in the west of Scotland around 1932 (T07-1930s), and another comprised a younger cohort of 1,444 participants who were born in the same region in 1952 (T07-1950s). Both groups completed an initial alcohol intake assessment in 1987-1988. Further intake assessments in 1990-1991 and 1995-1997 allowed the current study to establish longitudinal drinking trajectories for these participants. The current study sample comprised 869 of the T07-1930s' participants, and 1,002 of the T07-1950s' participants. For these participants, CHD events were identified through survey data and linkage to hospital record data and were coded using the Royal College of General Practitioners' codebook (codes 1940, 1945 and 1950). The mean follow-up time for incident CHD was 14.2 years for the T07-1930s' participants and 17.6 years for the T07-1950s' participants.

Whitehall II

The Whitehall II occupational cohort originally recruited participants from a pool of UK civil servants between 1985 and 1988. The recruited cohort comprised 10,314 participants. Of these, 8,573 met the inclusion criteria for the current study. Longitudinal alcohol intake was determined using reported consumption at the initial assessment in 1985-1988, and two further assessments in 1991-1994 and 1997-1999. Fatal and non-fatal CHD events and associated time-to-event data were obtained through linkage to national medical registry data. CHD events were classified according to the ICD codes for ischaemic heart disease: 410-414 (ICD-9) or I20-I25 (ICD-10). The mean follow-up time for incident CHD was 16.4 years.
